# Identification and external validation of a prognostic signature based on myeloid-derived suppressor cells-related LncRNAs to evaluate survival prognosis and treatment efficacy in invasive breast carcinoma

**DOI:** 10.1016/j.bbrep.2025.102261

**Published:** 2025-09-16

**Authors:** Yun Zhong, Tong Mu, Shunyao Zhang, Yuan Xiang, Lei Xie, Wenxiong Zhang, Kang Wang

**Affiliations:** a Department of Thoracic Surgery, The Second Affiliated Hospital, Jiangxi Medical College, Nanchang University, Nanchang, 330006, China; b The Second Clinical Medical School, Jiangxi Medical College, Nanchang University, Nanchang, 330088, China; c Department of Breast Surgery, The Second Affiliated Hospital, Jiangxi Medical College, Nanchang University, Nanchang, 330006, China; d Department of Traditional Chinese Medicine, The Second Affiliated Hospital, Jiangxi Medical College, Nanchang University, Nanchang, 330006, China

**Keywords:** Myeloid-derived suppressor cells, Long noncoding RNAs, Breast carcinoma, Prognostic signature, Bioinformatics, External validation

## Abstract

**Background:**

Originating in the hematopoietic tissue, myeloid-derived suppressor cells (MDSCs) significantly contribute to tumor-related immunological processes. However, their relationship with long noncoding RNAs (lncRNAs) and breast cancer remains incompletely understood. In this study, we introduced MDSCs-associated lncRNAs as novel prognostic biomarkers to assess outcomes in patients with invasive breast carcinoma (BRCA).

**Methods:**

Information regarding BRCA cases, including clinical and genomic details, was obtained from the TCGA repository. Predictive indicators were discovered, and their reliability underwent thorough verification. A clinically useful nomogram was developed following application-based validation. Additional investigations encompassed functional analysis, TMB assessment, TME profiling, immunotherapy efficacy forecasting, and drug sensitivity testing along with target identification. Long non-coding RNA expression was measured using reverse transcription quantitative PCR.

**Results:**

A risk stratification model incorporating eight MDSCs-related lncRNAs effectively predicted patient outcomes. Kaplan-Meier (K-M) survival analysis clearly indicated a much worse prognosis among patients classified as high-risk (p < 0.001). The nomogram accurately forecasted overall survival (OS). Analysis of functional enrichment revealed that pathways associated with epithelial cells showed activity among patients at higher risk. Characterization of the tumor microenvironment showed increased immune cell presence in those classified as low-risk. Conversely, individuals with greater risk displayed higher tumor mutational burden. TIDE and IPS analyses indicated superior immunotherapy responsiveness in the low-risk BRCA subgroup. Among 47 drugs with notable IC50 variations, Ribociclib, PD173074, KU-55933, NU7441, and nutlin-3a exhibited lower IC50 values within the low-risk group, whereas Lapatinib demonstrated greater efficacy among the high-risk group. Moreover, 10 potential therapeutic agents and their targets were predicted for high-risk patients. RT-qPCR validation confirmed the robustness of the model.

**Conclusions:**

We successfully verified a new model of molecular markers of MDSCs-related lncRNAs, offering critical insights for predicting outcomes and guiding therapeutic decisions in BRCA cases.

## Highlights

• First MDSC-related lncRNA prognostic model developed for BRCA patients.
• Risk score with 8 lncRNAs predicts prognosis and builds a nomogram.
• High-risk have high TMB and immunosuppression; low-risk have robust immunity.
• Drug analysis shows low-risk drugs effective; many agents predicted for high-risk.

## 1 Introduction

Invasive breast carcinoma (BRCA), the predominant malignancy among females, represents 32 % of cases in women [1]. Currently, BRCA is the second most prevalent cancer worldwide, second only to lung cancer [2]. This malignancy demonstrates significant invasiveness, possessing the ability to breach the basement membrane surrounding mammary ducts or lobular acinar structures. Such penetration into interstitial tissue elevates therapeutic challenges and complicates management approaches. Importantly, genetic tendencies and epigenetic influences frequently form the diversity of this illness [3]. Currently, TNM staging serves as a widely adopted criterion in clinical practice for BRCA. However, as precise personalized medicine advances, more precise evaluation methods, like biomarkers, are needed compared to TNM staging.

Long non-coding RNAs (lncRNAs), characterized by their length of at least 200 nucleotides and absence of protein-coding capabilities, have garnered significant interest in recent years. These molecules are increasingly investigated for their use as cancer markers in diagnosis and prognosis, and also as possible targets for treatment interventions [4,5]. These lncRNA-based models use specific biomarkers to predict disease progression and survival, improving prognostic accuracy and leading to personalized treatments. Nowadays, extensive research efforts have confirmed the effectiveness of lncRNA prediction models in cancer prognosis and demonstrated their usefulness in predicting patient prognosis.

Myeloid-derived suppressor cells (MDSCs) comprise a diverse group of myeloid lineage cells, including both myeloid progenitor cells and immature mononuclear cells [6]. These cells are recognized for their capacity to inhibit the immune system and have a critical function in various pathological conditions, especially in cancer [7]. Targeting MDSCs has emerged as a critical focus in advancing immunotherapy, offering promising potential to achieve novel therapeutic outcomes in this field.

Research has shown lncRNAs could offer new methods to alter the suppressive or overactive environment of diseases linked to MDSCs progression, including cancer [8,9]. Ample evidence suggests lncRNAs are crucial in the multiple stages of BRCA development [10]. We used MDSCs-related lncRNAs as a novel indicator to distinguish the risk among patients suffering from invasive BRCA. This research aims to investigate the potential of MDSCs-associated lncRNAs, laying the groundwork for more effective prognostic models and treatment strategies.

## 2 Methods

### 2.1 Data collection

In the primary phase of our investigation, we retrieved information concerning 1093 tissue specimens from the TCGA repository. This dataset comprised patient clinical records, unprocessed RNA sequencing results, and somatic mutation profiles. This was made possible through the utilization of the R package “TCGAbiolinks”, which was sourced from https://portal.gdc.cancer.gov/repository on September 8, 2024. Following the data acquisition, the mRNA data underwent a transformation process, converting it into TPM (Transcripts Per Million) format. Subsequently, a log2 conversion was applied for standardization purposes.

When selecting the samples, we adhered to specific criteria. Firstly, we focused on BRCA, encompassing all disease types. Secondly, we ensured the availability of comprehensive clinical and sequencing information, including details such as age, sex, stage, survival status, and more. To ensure the robustness of our findings, participants whose follow-up period did not exceed 30 days were systematically omitted from the dataset under examination.

To process RNA sequencing information formatted in FPKM, we utilized the Perl script, particularly Strawberry Perl 5.30.0, which was obtained from https://www.perl.org on September 8, 2024. Moreover, Perl enabled the retrieval of essential clinical details such as pathology, age, stage, and survival statistics from the database.

### 2.2 Select MDSCs-associated genes and MDSCs-related lncRNAs

From previous investigations, we select 52 genes linked to MDSCs [[7], [8], [9], [10]]. Furthermore, we explored the relationships between MDSCs-associated genes and all lncRNAs using Pearson correlation analysis. By using a coefficient threshold above 0.3 and p-value under 0.05, we identified lncRNAs linked to MDSCs [11]. We then conducted analysis of variance on data from the corr[elation study, applying "DESeq2" [12] computational tool. Through this process, lncRNAs meeting specific statistical thresholds (p < 0.05) and demonstrating substantial expression alterations (|log2FC| > 3) were identified.

### 2.3 Construct BRCA MDSCs-associated lncRNA prognostic model

The MDSCs patient group underwent random allocation to establish training and test groups (1:1). The model was constructed using the training cohort, while validation was performed by the test cohort. Patient characteristics in the TCGA database are listed in Table 1.

**Table 1:** Clinical information of train, test, entire groups.

Characteristics	Train cohort(n = 547)		Test cohort(n = 546)		Entire cohort(n = 1093)	
	n	%	n	%	n	%
**Age**						
<60	283	51.74	283	51.83	566	51.78
≧60	264	48.26	264	48.35	528	48.31
**Gender**						
Female	543	99.27	538	98.53	1081	98.9
Male	4	0.73	8	1.47	12	1.1
**Status**						
Alive	471	86.11	473	86.63	944	86.37
Dead	76	13.89	73	13.37	149	13.63
**Stage**						
Stage I	89	16.27	93	17.03	182	16.65
Stage II	322	58.87	297	54.4	619	56.63
Stage III	119	21.76	129	23.63	248	22.69
Stage IV	8	1.46	12	2.2	20	1.83
Stage X	6	1.1	7	1.28	13	1.19
unknow	3	0.55	8	1.47	11	1.01
**T Stage**						
T1	120	21.94	138	25.27	258	23.6
T2	319	58.32	314	57.51	633	57.91
T3	65	11.88	73	13.37	138	12.63
T4	21	3.84	18	3.3	39	3.57
TX	0	0	3	0.55	3	0.27
**M Stage**						
M0	466	85.19	443	81.14	909	83.17
M1	10	1.83	12	2.2	22	2.01
MX	71	12.98	91	16.67	162	14.82
**N Stage**						
N0	253	46.25	262	47.99	515	47.12
N1	193	35.28	168	30.77	361	33.03
N2	55	10.05	65	11.9	120	10.98
N3	35	6.4	42	7.69	77	7.04
NX	11	2.01	9	1.65	20	1.83

**Abbreviations:** T stage: Tumor stage; N stage**:** Node stage; M stage**:** metastasis stage.

By employing the "survival" package, MDSCs-associated lncRNAs linked to BRCA patient prognosis were identified via univariate COX analysis (p < 0.05). To further narrow down and identify the most predictive MDSCs-related lncRNAs, LASSO regression combined with multivariate COX regression analysis was applied. Graphical representation of analytical models was generated through employment of computational tools, specifically "survival", "survminer", "glmnet", and "timeROC". For every BRCA case, a prognostic score associated with MDSCs was derived through mathematical computation: risk score = ∑ (Coefi ∗ Expi), where Coefi signifies weighting factors and Expi represents expression values for individual lncRNAs.

Variations in OS and PFS across various cohorts were assessed by utilizing the Kaplan-Meier (K-M) approach, implemented with "survival" as well as "survminer" tools throughout the entire study population. Additionally, we calculated hazard ratios (HRs) along with log-rank p-values and corresponding 95 % confidence intervals (CI) for evaluating the statistical relevance of differences in patient survival across categories.

To ensure accuracy, Principal Component Analysis (PCA) [13] was applied to multiple transcriptomic datasets, encompassing complete gene collections, total lncRNAs, MDSCs-associated transcripts, and markers identified for predictive models. This approach verified the robustness of classification.

### 2.4 Independent prognostic analysis

To reduce interference from additional clinical factors, survival outcomes were stratified according to patient characteristics, focusing on age, sex, and tumor stage. Subsequently, COX single and multi-factor evaluations examined if MDSCs-linked lncRNA signs might separately anticipate prognosis.

### 2.5 Nomogram development and assessment in BRCA

To develop the nomogram, we analyzed ROC values at 1, 5, and 7 years along with clinical factors, computing the C-index for assessing model prediction using the "survminer," "survival," "timeROC," "pec," and "rms" packages.

Using computational tools such as "survcomp", "survival", "regplot", and "rms", prognostic charts were developed to estimate BRCA patients' survival probabilities at 1, 5, and 7 years. These nomograms incorporated risk score features and clinicopathological elements. To assess how well the nomogram predicts compared to our risk model, decision curve analysis (DCA) was conducted. Additionally, thorough assessments were done on these prediction tools. For consistency evaluation, calibration plots were used to contrast survival probabilities from our model with actual patient outcomes.

### 2.6 Enrichment analysis

To uncover potential pathways and functional associations linked to this gene set, we employed Gene Ontology (GO) enrichment analysis, Kyoto Encyclopedia of Genes and Genomes (KEGG) pathway and functional enrichment analysis, along with Gene Set Enrichment Analysis (GSEA). Specifically, enrichment analysis was conducted for eight gene sets using the GSEA database (https://www.gsea-msigdb.org), including: Positional Gene Sets (c1), Curated Gene Sets (c2), Motif Gene Sets (c3), Computational Gene Sets (c4), Gene Ontology Gene Sets (c5), Cancer Gene Sets (c6), Immunologic Signatures Gene Sets (c7), Chemical and Genetic Perturbations Gene Sets (c8).

Utilizing the simulation framework of 8 MDSCs-associated lncRNAs, cases were categorized as high or low risk, with clinical details shown in Table 2. After this classification, enrichment analysis identified metabolic pathways and functions with statistical significance, indicated by p-values under 0.05.

**Table 2:** Clinical information of low risk and high risk groups.

Characteristics	Low-risk group(n = 531)		High-risk group(n = 562)	
	n	%	n	%
Age				
<60	284	53.48	304	54.09
≧60	247	46.52	258	45.91
**Gender**				
Female	528	99.44	553	98.4
Male	3	0.56	9	1.6
**Status**				
Alive	489	92.09	455	80.96
Dead	44	8.29	107	19.04
**Stage**				
Stage I	105	19.77	77	13.7
Stage II	292	54.99	327	58.19
Stage III	116	21.85	131	23.31
Stage IV	8	1.51	12	2.14
Stage X	6	1.13	7	1.25
unknow	4	0.75	7	1.25
**T Stage**				
T1	164	30.89	116	20.64
T2	285	53.67	348	61.92
T3	67	12.62	71	12.63
T4	15	2.82	24	4.27
TX	0	0	3	0.53
**M Stage**				
M0	434	81.73	475	84.52
M1	9	1.69	13	2.31
MX	88	16.57	74	13.17
**N Stage**				
N0	254	47.83	261	46.44
N1	183	34.46	178	31.67
N2	50	9.42	70	12.46
N3	38	7.16	39	6.94
NX	6	1.13	14	2.49

**Abbreviations:** T stage: Tumor stage; N stage**:** Node stage; M stage**:** metastasis stage.

### 2.7 Assessment of TME and immune infiltration

The 'ESTIMATE' R tool gauged immune shifts, stromal amounts, tumor distinctness, and total ESTIMATE figures among risk cohorts. Additional insights were gathered from the Tumor Immune Estimation Resource (TIMER) 2.0 platform (accessible at http://timer.cistrome.org/until September 11, 2024).

TME (Tumor Microenvironment) score offers a valuable depiction of disparities in stromal and immune cells across different risk groups. To detail the constitutive shifts in immune cells, Single-sample Gene Set Enrichment Analysis (ssGSEA) was used to assess the infiltration levels of 22 distinct immune cell types.

### 2.8 Analysis of TMB

To examine the frequencies of somatic mutations across different risk cohorts, genomic alteration information obtained from TCGA was processed and evaluated with the MAFTOOLS software [14]. We chose the genes exhibiting the highest mutation rates and visualized them using a waterfall plot. Concurrently, we are exploring the correlation between survival rates, risk scores, and tumor mutational burden (TMB). Additionally, to conduct more nuanced analyses, we merged TMB status and risk profiles.

### 2.9 Evaluation of TIDE and IPS

Tumor Immune Dysfunction and Exclusion (TIDE) functions as an algorithmic approach to assess the potential of immune evasion in malignancies and predicting responses to immunotherapeutic interventions. To comprehensively analyze differences among BRCA cases stratified by risk levels associated with immune evasion and exclusion, we extracted TIDE results from its online database (available at http://tide.dfci.harvard.edu/until September 12, 2024) [15].

Based on machine learning, the Immunophenoscore (IPS) generates z scores through the assessment of four cellular components linked to immunogenicity. Studies have demonstrated that IPS can effectively assess tumor immunogenicity and forecasts immune checkpoint inhibitor (ICI) therapy in various tumor types. For BRCA patients, IPS data were sourced from The Cancer Immunome Atlas (TCIA) [16]. (accessible at https://tcia.at/home until September 12, 2024).

### 2.10 Prediction of drugs’ IC50 and CMap analysis

The pharmaceutical agents utilized to discern varying IC50 levels between different risk cohorts were obtained from the Genomics of Drug Sensitivity in Cancer (GDSC) platform (accessible at https://www.cancerrxgene.org/until September 13, 2024) [17]. For the purpose of assessing the applicability of our model in clinical decision-making, the "pRophetic" tool was utilized to determine the IC50 levels for frequently administered anticancer medications. A lower IC50 means better treatment. We compared IC50 among different risk cohorts using the limma and ggpubr packages. And we have identified pathways associated with these drugs.

Furthermore, to identify potential therapeutic agents, we queried the Connectivity Map database with differentially expressed genes linked to the MDSCs-related risk signature (accessible at https://clue.io/until September 15, 2024). This evaluation produced correlation values between −100 and 100, determined by the abundance of differentially expressed genes [18,19]. For therapeutic interventions in high-risk patients, we identified 11 compounds (with correlations below −75), including their targets and Mechanism of Action (MOA).

### 2.11 Validation via RT-qPCR

The non-cancerous mammary epithelial cell line, MCF-10A, together with the malignant breast cell lines, MCF-7 and T47D, were obtained from Sigma-Aldrich, a division of Merck KGaA located in Darmstadt, Germany. MCF-10A cultures were maintained in Mammary Epithelial Cell Growth Medium (MEGM) sourced from Lonza Group AG in Basel, Switzerland. In contrast, MCF-7 and T47D were cultivated in a solution containing 90 % Dulbecco's Modified Eagle Medium (DMEM) from Gibco, part of Thermo Fisher Scientific in Waltham, MA, USA, and 10 % fetal bovine serum. Total RNA extraction was performed with Trizol reagent from Thermo Fisher Scientific. cDNA production was achieved through a reverse transcription kit also from Thermo Fisher Scientific. Following this, RT-qPCR was carried out using the SYBR Green premixed qPCR kit from the same company, with reactions conducted on a Roche LightCycler 480 II system, provided by Roche in Basel, Switzerland. Transcript abundance was determined by the 2^−ΔΔCt approach, and the primer sequences can be found in Table S1.

To evaluate protein expression levels of the target genes within normal breast tissues and BRCA tissues, especially concerning MDSCs, the Human Protein Atlas database was utilized.

## 3 Results

### 3.1 Screening and risk model construction of MDSCs related lncRNAs

**Fig. 1:**
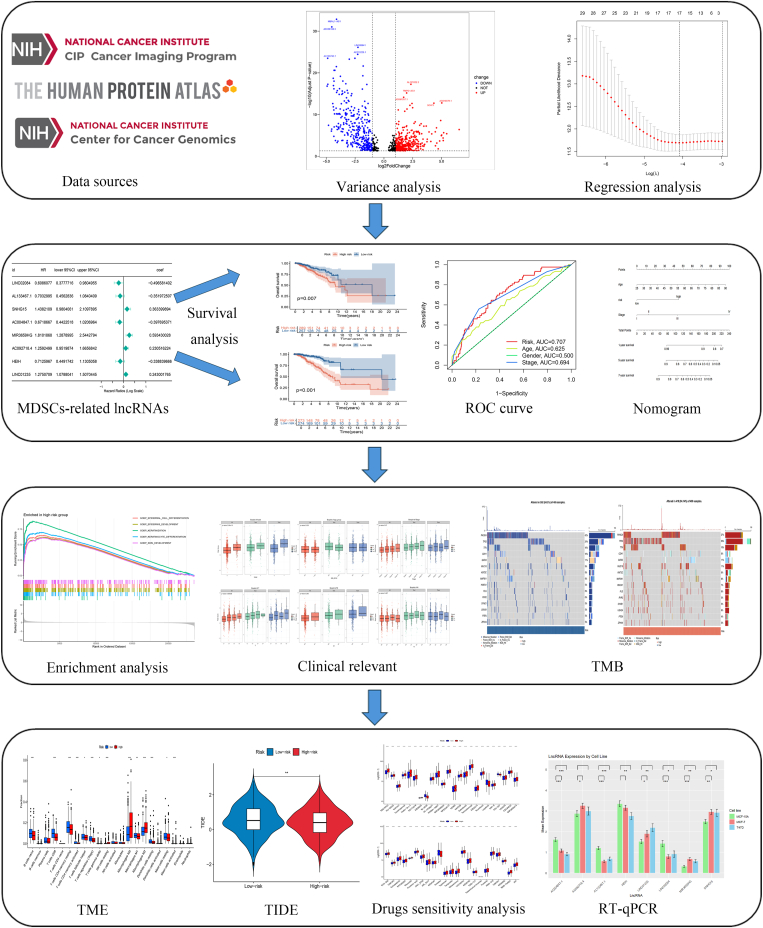
Flow chart.

Fig. 1 illustrates the flowchart of our research. Data from RNA-seq experiments for BRCA cases were obtained from TCGA, including mRNA expression datasets, and 16,882 lncRNAs were detected after correction and normalization steps. Among these, 52 genes associated with MDSCs were pinpointed. A network of protein-protein interaction (PPI) involving 36 genes was built using the STRING platform (Fig. S1B). Differentially expressed lncRNAs were detected using thresholds of |Log2 FC| > 1, p < 0.05, correlation coefficients >0.4, and p < 0.001. Following this rigorous screen process, 209 lncRNAs associated with MDSCs were ultimately selected. The coexpression network involving MDSCs-associated genes and lncRNAs is displayed in Fig. S1C.

The TCGA database was utilized to extract clinical and survival data pertaining to BRCA patients. Among the 35 MDSCs-related lncRNAs screened (Fig. S1A), we identified 17 MDSCs-related lncRNAs of BRCA patients by using univariate Cox regression analysis. Thereafter, we utilized LASSO filtering out 14 lncRNAs (Fig. S1D and E) and obtained their coef in LASSO (Table S2). multifactorial COX analyses and finally filtered out 8 key lncRNAs and gain their final coef (Fig. S2A) for constructing the prognostic model. The correlation heatmap between these 8 lncRNAs and MDSCs genes is in Fig. S2B. And we also found that among these 8 lncRNAs, AC004847.1, LINC02084 and AL 133467 have the highest positive correlation property with each other (Fig. S2C). These 8 lncRNAs were employed to determine risk scores for individual BRCA cases.

The risk score = $\sum_{i=1}^{8}\left(\text{coefficient}*\text{expression}\right)\;$ = (−0.496581402 ∗ LINC02084) + (−0.351972507 ∗ AL133467.1) + (0.363399894 ∗ SNHG15) + (−0.397695370 ∗ AC004847.1) + (0.593430028 ∗ MIR3659HG) + (0.230516224 ∗ AC092718.4) + (−0.338839666 ∗ HEIH) + (0.243001765 ∗ LINC01235)

### 3.2 Development and verification of the prognostic model

For guaranteeing the precision of our classification, survival rates among these groups were analyzed across the training, test, and combined sets. Within the test, training, and PFS groups, high risk patients consistently showed reduced survival compared to low risk individuals (Fig. S3A–D). The concordance index curve is in Fig. S4C.

**Fig. 2:**
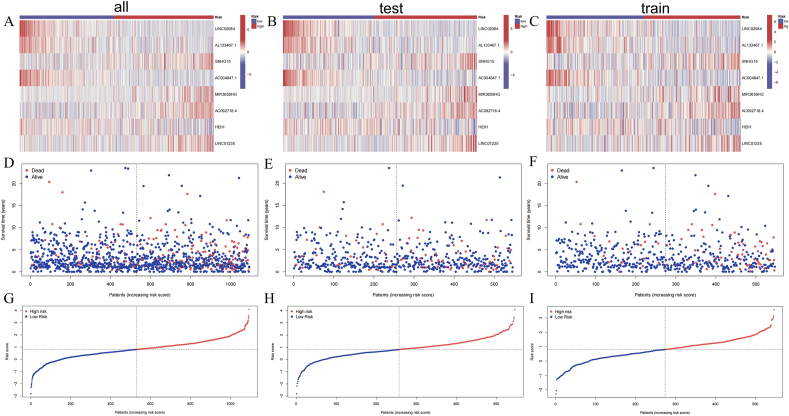
Model prediction accuracy is validated across entire, test and train group. (A–C) Heatmaps that show 8 MDSCs-related lncRNAs expressions; Scatterplots (D–F) and Risk curves (G–I) which visualize patient survival status.

Expression profiles of eight MDSCs-linked lncRNAs in high- and low-risk categories are visualized through heatmaps for the training, test, and overall cohorts. Notably, higher expression levels of LINC02084, AL133467.1, AC004847.1, and HEIH are mainly observed in low-risk patients, while SNHG15, MIR3659HG, AC092718.4, and LIC01235 show increased abundance in high-risk individuals (Fig. 2A–C). Additionally, Fig. S4B provides a detailed depiction of the correlations among risk scores, various clinical characteristics, and the eight lncRNAs included in our models. The scatterplots and risk surves show that patients who had higher risk scores faced a greater chance of unfavorable survival outcomes, whereas the majority of survival cases were predominantly found in subgroup with low risk scores, which validate the prognostic (Fig. 2D–I). The relationships between different clinical data and risk score among entire group, test group and train group are shown in Fig. S5. We found that p-values of fustat and T-stage are lower than 0.05.

We also validated our model by PCA. Compared with the group of all genes (Fig. S6B), the lncRNA group of MDSCs (Fig. S6C) and the gene group of MDSCs (Fig. S6D), we found that the risk classification group we constructed (Fig. S6A) was obviously more effective.

### 3.3 Analysis of independent factors of MDSCs-related lncRNAs in BRCA

To evaluate the impact of clinicopathological factors on prognostic assessments, the predictive utility of our risk model was examined within diverse subgroups stratified by age and disease stage. Results demonstrate that patients categorized as high risk exhibited poorer OS relative to their low-risk counterparts in every clinical subgroup. (Figs. S7A, B, D, E).

We compared the risk model's prognostic value among diverse clinicopathological groups of BRCA, considering age, gender, and T-stage. t demonstrates that univariate COX regression analysis revealed a strong link between OS in BRCA patients and factors like age, stage, and risk score (Fig. S7F). Multivariate COX regression analysis determined age, disease stage, and risk score as independent prognostic factors (Fig. S7C). Furthermore, high-risk individuals consistently exhibited poorer Overall Survival in relation to low-risk cases throughout every clinical group analyzed.

### 3.4 Development of a prognosis nomogram

**Fig. 3:**
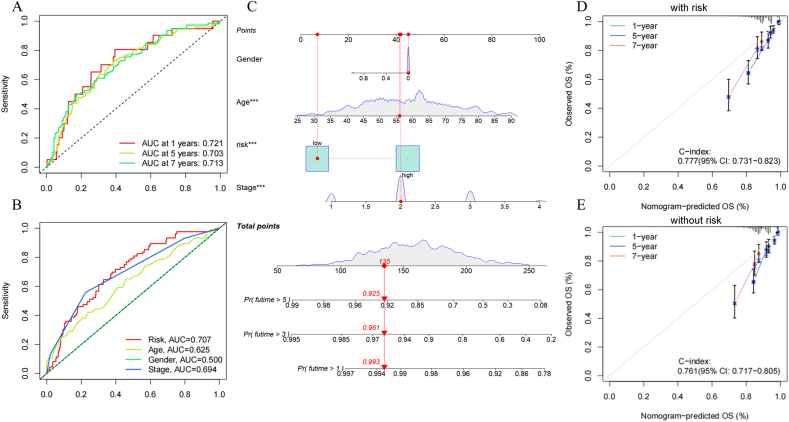
ROC curves and construction of prognostic nomogram. (A) ROC curves for 1-year, 5-year, 7-year periods. (B) Comparing ROC curves of predictive risk models with clinicopathological characteristics. (C) A nomogram predicting the survival time 1, 5, 7 years of these patients we selected. (D) The calibration curves (with risk). (E) The calibration curves (without risk).

The ROC curve validated the diagnostic precision of the MDSCs-associated lncRNA model in assessing 1-, 5-, and 7-year OS, achieving AUC values of 0.721, 0.703, and 0.713 (Fig. 3A). Moreover, the risk score's predictive performance (AUC = 0.707) significantly exceeded that of other clinical variables, such as gender, age, and stage (AUC = 0.500, 0.625, 0.694) (Fig. 3B). In our model, clinical factor gender has no signification. This may be caused by the limited number of male samples.

To integrate the risk model into clinical settings, we constructed a prognostic line graph model tailored to MDSCs. This model facilitates the estimation of OS rates at 1, 5, and 7 years, respectively. This model provides a visual representation of prognostic outcomes of the samples we selected. Among these factors, we found that Gender had no significant effect on OS (Fig. 3C). Additionally, calibration curves reveal strong agreement between our model's predictions and actual survival probabilities at these time points, surpassing those without our risk model (Fig. 3D and E). In Decision Curve Analysis (DCA) curve, nomoscore with our risk model is guaranteed to be effective at a high-risk threshold of 0.1–0.7. And the effect of nomoscore with our risk model is much greater than that of other factors, including nomoscore without our risk model (Fig. S5C). These findings underscore the reliability and stability of the MDSCs-related lncRNA prognostic signature, indicating its utility in estimating survival time and guiding clinical treatments for BRCA patients.

In summary, we constructed a new empty nomogram that excludes gender, a nonsensical clinical factor, in order to accurately predict OS in the clinic (Fig. S4D).

### 3.5 Enrichment analysis

**Fig. 4:**
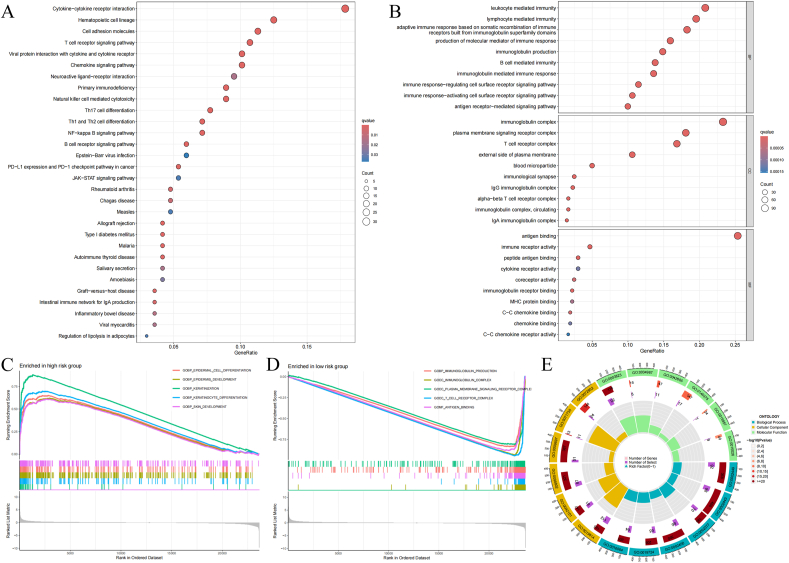
enrichment analysis. (A) The Bubble chart of KEGG between different risk groups. (B) GO Enrichment bubble chart in different groups. (C) Charts of c5 of GSEA enrichment analysis in high risk group. (D) Charts of c5 of GSEA enrichment analysis in low risk group. (E) GO Enrichment Circle Plot in different risk groups.

KEGG enrichment analysis revealed substantial enrichment in numerous immune-related pathways, such as T cell receptor interaction, cytokine-cytokine receptor interaction, differentiation of Th1, Th2, and Th17 cells, NF-kappa B as well as B cell receptor molecular routes, among others (Fig. 4A).

Furthermore, GSEA was performed to explore essential variations in biological mechanisms and signaling networks between risk groups stratified by the 8 MDSCs-associated lncRNAs signature. When we focused on Gene Ontology Gene Sets (c5), it was observed that among the elevated-risk subgroup, functions and biological routes pertinent to with epidermis development are predominantly enriched (Fig. 4C). Then in low-risk subgroup, the enriched results are tended to connect with immune pathways and functions, including immunoglobulin production, immunoglobulin complex, antigen binding and T cell receptor complex. It's worth noting that plasma membrane signaling receptor complex was also selected (Fig. 4D). The results of other gene sets are in Fig. S8. The whole information from all the gene sets in GSEA is shown in Table S3. Gene Ontology (GO) enrichment analysis revealed the enrichment of biological processes (BP), molecular functions (MF), and cellular components (CC). The details of outcomes in these three assessments are shown in Fig. 4, Fig. 7E.

### 3.6 Tumor immune microenvironment in different groups

**Fig. 5:**
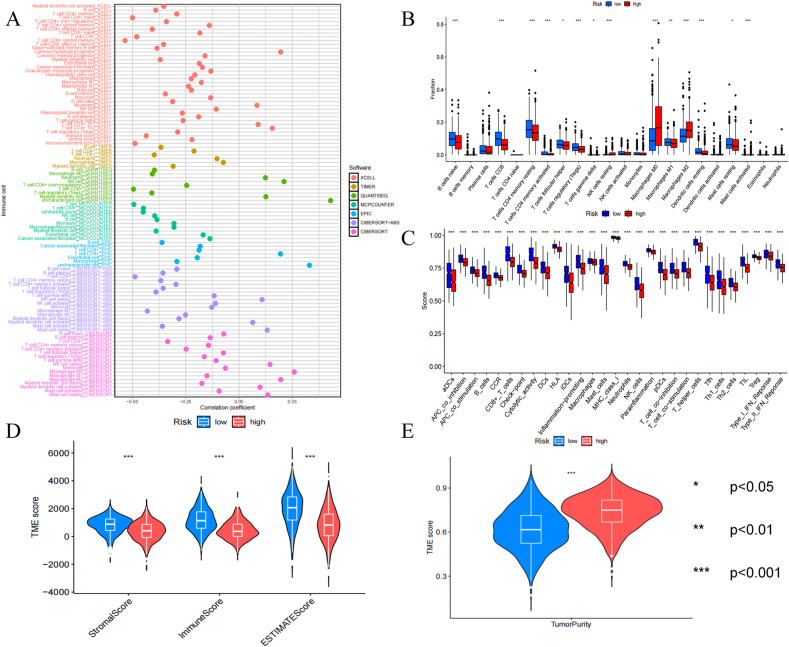
Tumor immune microenvironment analysis. (A) The Bubble chart for proportions of 22 immune cells under CIBERSORT algorithm (B) Box plot of immune cells infiltration between high and low group. (C) Box plot of the scores of immune cells ratios and immune-related functions. (D, E) A vioplot of stromalscore, immunescore, ESTIMATEscore and TumorPurity between high and low group.

We found that immune-related mechanisms predominantly drove the differences distinguishing various risk groups through enrichment analysis. Subsequently, tumor immune context was analyzed to probe shifts in immune cell levels within these sets. The connection linking immune cells to risk scores was determined through multiple methodologies (Fig. 5A). The findings demonstrated that all exhibited elevated levels in the decreased-hazard category relative to the increased-hazard category, except for tumor purity (Fig. 5D,E).

Additional analysis in Fig. 5B uncovered notable variations in immune cell infiltration within TME. The low-risk category exhibited increased levels of infiltration for multiple immune cell populations, including naive B cells, CD8 T cells, resting and activated CD4 memory T cells, follicular helper T cells, Tregs, gamma delta T cells, M1 macrophages, resting dendritic cells, and resting mast cells. In contrast, the high-risk subgroup displayed higher infiltration levels in resting NK cells, M0 and M2 macrophages, and activated mast cells. Risk scores and 14 distinct immune cells are depicted in Fig. S9. Further ssGSEA analysis emphasized substantial variations in immune-related processes and cell proportions between the two risk categories (Fig. 5C).

### 3.7 Tumor mutation landscapes

**Fig. 6:**
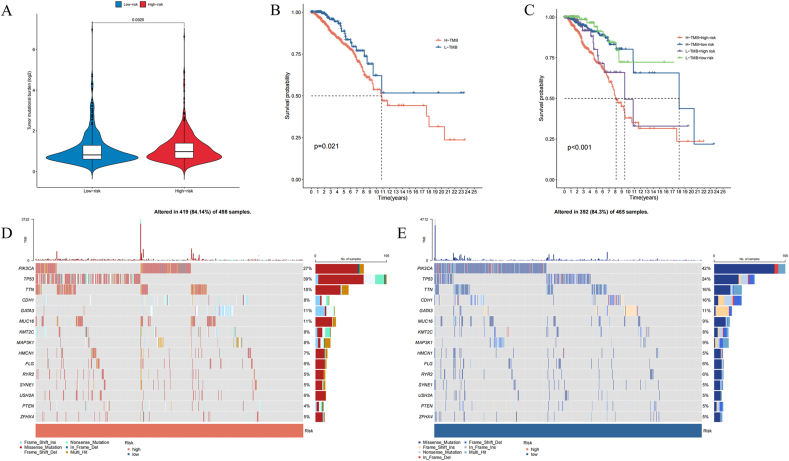
Tumor mutation landscapes of our signature. (A) A vioplot in different risk groups. (B) The K-M curves show survival status and time in different TMB groups. (C) K-M curves for Stratified survival analysis. (D, E) Waterfall charts showing the mutants status in the high and low risk groups.

We studied tumor mutation burden (TMB) across different groups based on MDSCs-related lncRNAs. Findings suggest that the high-risk category displays a markedly elevated TMB relative to the low-risk category (Fig. 6A). Correspondingly, Fig. 6B shows that patients with low TMB exhibit improved survival probabilities compared to those with high TMB. Stratified survival analysis further indicates that individuals with both low risk and low TMB achieve the most favorable survival outcomes among all groups (Fig. 6C). We also examined the top 15 mutated genes in various risk categories. PIK3CA, TP53, TTN, CDH1, and GATA3 emerged as genes with the highest mutation rates. TTN and GATA3 demonstrate comparable mutation frequencies. TP53 mutations are more prevalent in the high-risk individuals, whereas PIK3CA and CDH1 changes prevail in the low-risk cluster (Fig. 6D and E).

**Fig. 7:**
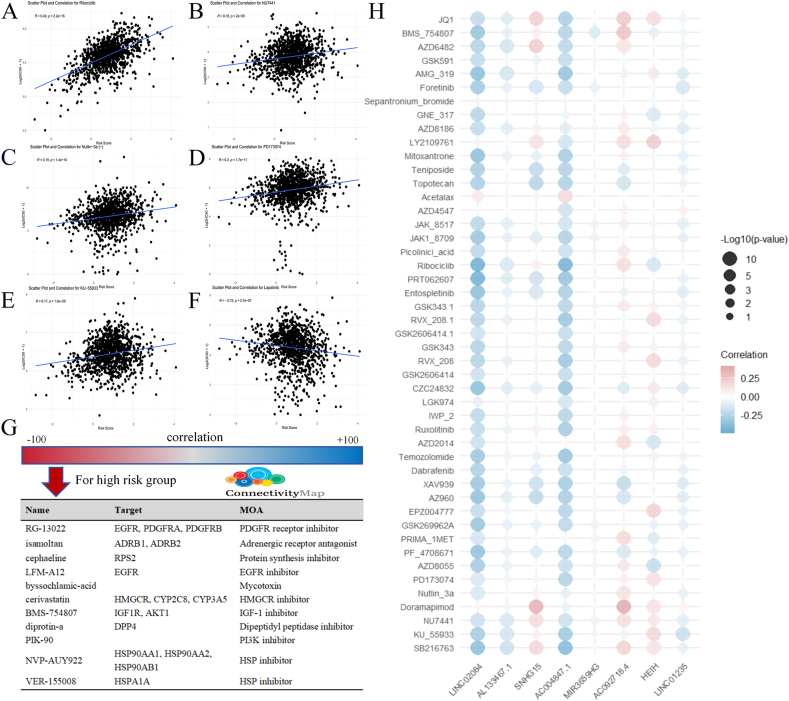
Prediction of drugs' IC50 and potential drug targets. (A) Ribociclib. (B) NU7441. (C) nutlin-3a. (D) PD173074. (E) KU-55933. (F) Lapatinib. (G) Drugs with their targets and MOA for high risk group from CMap analysis. (H) A bubble chart of the correlation between our model lncRNA and 47 drugs with p-value less than 0.001.

### 3.8 Prediction of immunotherapy response

TIDE evaluates immune checkpoint therapy reactions by integrating TIDE and IFNG markers. The share of FALSE No-benefits rose significantly in the low-risk set versus the high-risk cluster (Fig. S10A). Moreover, the low-risk set displayed heightened immune impairment, while the high-risk cluster revealed greater immune suppression (Fig. S10B and S10C). Additionally, immune dysfunction was intensified in the low-risk group, while immune exclusion increased in the high-risk group (Fig. S10D and S10F). Furthermore, the low-risk group showed increased immune dysfunction, while the high-risk group exhibited higher immune exclusion.

### 3.9 Prediction of drugs’ IC50 and potential drug targets

Ninety-four compounds exhibited significant IC50 differences (p < 0.05) between different risk categories (Fig. S11). These drugs have basically been confirmed to be linked to BRCA in studies, and the IC50 of most of them increases with the increase of risk score, such as: Ribociclib, PD173074, KU-55933, NU7441 and nutlin-3a (Fig. 7A,D); the IC50 of a small part of them decreases within risk score's increasing, such as Lapatinib (Fig. 7E,F). Specifics of these 47 medications appear in Table S4. Additionally, we also list details of the 46 drugs with p-value ranging from 0.001 to 0.05 in Table S5. In these two tables, we found that there are more drugs targeting pathways DNA replication and PI3K/MTOR signaling. Furthermore, we used CMap to predict drugs with their targets and Mechanism of Action (MOA) that are more potentially beneficial in high-risk patients. The MOA include HSP inhibitor, PI3K inhibitor, Dipeptidyl peptidase inhibitor, IGF-1 inhibitor, HMGCR inhibitor, Mycotoxin, EGFR inhibitor, Protein synthesis inhibitor, Adrenergic receptor antagonist and PDGFR receptor inhibitor (Fig. 7G). These targets and MOA can guide the development of future drugs for BRCA. We next produced a bubble graph linking our model's lncRNA to 47 drugs, p-values below 0.001 among risk sets, highlighting LINC02084 and AC004847.1 as strongly opposed to most agents (Fig. 7H). Drugs with p-value above 0.05 are listed in Table S6.

### 3.10 Validation of risk models through In vitro experiments

**Fig. 8:**
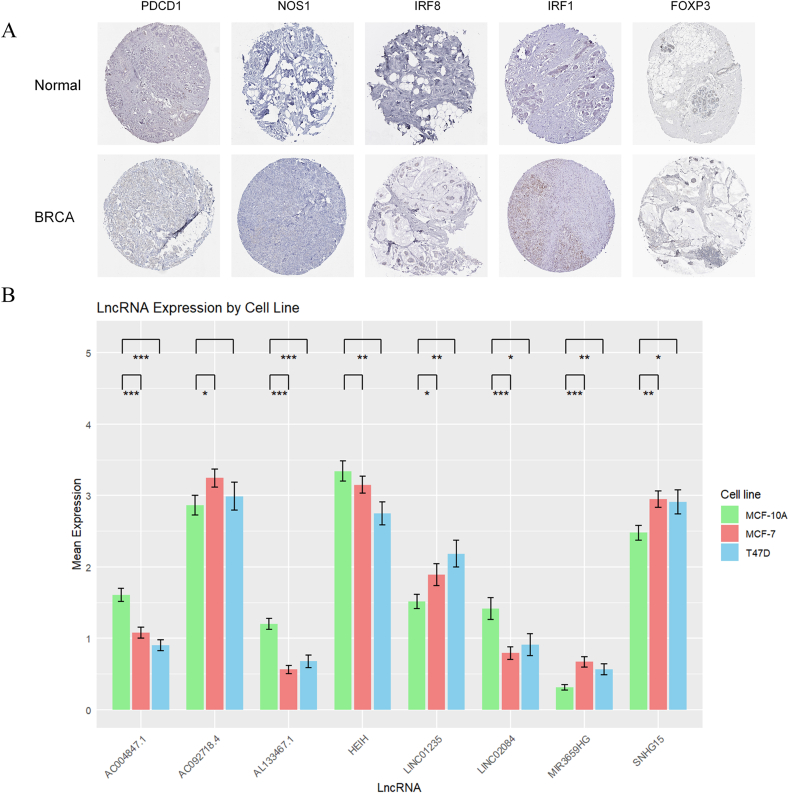
In vitro experimental validation of the risk model. Immunohistochemical staining images of partial MDSCs-associated gene proteins in BRCA tissue and normal tissue (A); Relative expression of 9 MDSCs-related lncRNAs in different risk subgroups (B). ∗p < 0.05, ∗∗p < 0.01, ∗∗∗p < 0.001**.**

Staining results from HPA data served to show protein quantities of picked MDSCs-related genes in BRCA against normal specimens (Fig. 8A).

The RT-qPCR findings indicated that out of the 8 MDSCs-related lncRNAs, AC004847.1, AL133467.1 and LINC02084 showed elevated expression in MCF-10A cells, whereas LINC01235, MIR3659HG and SNHG15 demonstrated higher expression levels in MCF-7 and T47D cells. There is no obvious difference between AC092718.4 in MCF-10A and T47D cell lines, and at the same time, there is no obvious difference between HEIH in MCF-10A and MCF-7 cell lines (Fig. 8B).

## 4 Discussion

Breast cancer remains a leading global health burden, ranking second in cancer incidence after lung cancer [1]. The exploration of prognostic biomarkers involving lncRNAs, has garnered much attention in recent years because of their potential to elucidate the complex molecular mechanisms underlying cancer development and progression. Prior research has ventured into the prognostic value of lncRNAs across various cancer types, including BRCA [4,5]. However, a significant limitation of these studies is that they often overlook the specific biological roles and complex interactions of lncRNAs with key immune cells, such as MDSCs. Our study addresses this knowledge gap by adopting a targeted approach, focusing specifically on MDSCs-related lncRNAs.

In our study of BRCA, we identified eight lncRNAs associated with MDSCs that are significantly correlated with BRCA prognosis. These lncRNAs were incorporated into a risk score model. Notably, in our model, LINC02084 and AC004847.1 exhibited negative correlations with most drugs, with p-values less than 0.001, alongside strong positive correlations, suggesting a potential underlying connection between them. However, there are currently no studies on how these two lncRNAs produce correlations, which could point to a new direction for future research. Based on these eight lncRNAs, we successfully constructed an effective reference nomogram to predict patients' overall survival (OS) in BRCA, producing strong AUC results across various year predictions. The accuracy of this performance exceeds that of established models, such as the Breast Cancer Risk Assessment Tool (BCRAT) and the Breast Cancer Surveillance Consortium (BCSC), which achieve a maximum AUC of only 0.71 [20].

In the cohort categorized as low-risk, pathways associated with the immune system, including interactions between cytokines and their receptors, signaling of T cell receptors, and differentiation of Th1, Th2, and Th17 cells, are notably enriched, indicating a heightened immune reactivity [21]. Particularly, this group exhibits increased levels of infiltration by several types of immune cells, CD8^+^ T cells can directly kill tumor cells, while CD4^+^ T cells enhance the persistence of immune responses, and B cells are involved in antigen recognition and immunoglobulin production [22]. In contrast, the high-risk set features abundant immune-suppressive cells, including M2 macrophages and resting NK cells, which are associated with immune evasion and tumor progression. M2 macrophages promote tumor growth by secreting anti-inflammatory cytokines, while resting NK cells may not effectively contribute to anti-tumor immunity. Furthermore, patients in the high-risk category demonstrate elevated tumor purity and reduced immune and stromal scores, suggesting a microenvironment less favorable for immune cell activity, which may lead to a worse prognosis. Additionally, the high-risk subgroup is characterized by a higher TMB, which often leads to immune tolerance and evasion, weakening the immune capacity to attack tumor cells [23]. Elevated TMB can result in altered antigen presentation and a diminished response immune checkpoint therapy. Interestingly, the low-risk cohort shows a favorable response to ICIs, likely due to its more active immune profile. Collectively, these immune and genomic characteristics suggest the low-risk subgroup benefits from a more effective immune surveillance system, while the high-risk group is hampered by immune suppression, immune tolerance, and a higher TMB, leading to poorer survival outcomes.

We identified 47 antitumor drugs related to BRCA that could potentially be more effective in different risk groups, such as Lapatinib [24], Ribociclib [25], PD173074 [26], KU-55933 [27], NU7441 [28], and nutlin-3a [29]. These drugs largely target critical cellular processes such as DNA replication and the PI3K/MTOR signaling pathway, both of which are essential for tumor progression [30]. Drugs such as Ribociclib and Nutlin-3a, which regulate cell cycle checkpoints and apoptosis, revealed raised IC50 figures in the high-risk cluster, suggesting resistance in these cases, likely due to alterations in cell cycle regulation and apoptotic pathways, thus requiring higher doses or combination therapies. Conversely, Lapatinib, an EGFR and HER2 inhibitor, demonstrated a reduced IC50 in the high-risk subgroup, implying that it might exhibit greater efficacy in this cohort, possibly due to its impact on pathways such as EGFR and HER2 signaling, which could be more active in high-risk tumors. The increased sensitivity to several drugs in the low-risk group could be attributed to better-functioning apoptotic pathways and less dysregulated cell cycle checkpoints, facilitating more effective responses to these agents. The prevalent enrichment of the DNA replication and PI3K/MTOR signaling pathways across both risk groups is particularly notable, as these pathways are critically involved in tumor cell proliferation, survival, and resistance to treatment. Dysregulation of PI3K/MTOR signaling has been extensively studied in BRCA, with mutations in PI3KCA and alterations in PTEN commonly observed in tumors with poor prognosis. Drugs targeting the PI3K/MTOR pathway, such as Everolimus and Idelalisib, have been explored in clinical trials with varying degrees of success [31].

The CMap analysis, which identified additional drugs and their MOA, revealed several potential therapeutic candidates, including HSP inhibitors, PI3K inhibitors, and EGFR inhibitors, which may be particularly beneficial for high-risk patients. Heat shock protein (HSP) inhibitors are emerging as promising cancer therapeutics due to their ability to disrupt the protein homeostasis machinery that tumors rely on for survival, especially under stress conditions. Emerging research has underscored the significance of HSP90 inhibitors, including Ganetespib, in addressing therapeutic resistance across multiple cancer types, particularly BRCA [32]. PI3K inhibitors, which have been studied extensively for their role in suppressing tumor growth, also present a viable strategy, with Alpelisib showing efficacy in tumors with PI3K pathway mutations [33]. In addition, the identification of Adrenergic receptor antagonists and Mycotoxins as potential therapeutic agents in the high-risk group opens up new avenues for research. Adrenergic receptors, particularly β-adrenergic receptors, have been implicated in cancer progression by enhancing tumor cell migration, invasion, and metastasis [34].

The prognostic model developed in this study can aid in early risk stratification of BRCA patients, facilitating timely and appropriate treatment decisions. By identifying high-risk patients, clinicians can initiate more aggressive therapy or closer monitoring, potentially improving patient outcomes. Prediction of drugs’ IC50 and potential drug targets based on our model offers valuable insights into personalized treatment strategies. Tailored drug selection can improve therapeutic efficacy, reducing unnecessary exposure to ineffective or harmful agents. Our findings on immune infiltration and TIDE & IPS indicate that low-risk individuals could thrive more with immunotherapy. This information can guide immunotherapy decisions, maximizing response rates and survival benefits. And our results are consistent with previous findings [35]. However, this investigation is constrained by some limitations, including sample size and generalizability. This study was based on data from TCGA, which may limit its generalizability to other patient populations. Further validation in larger, more diverse cohorts is necessary to confirm our findings.

## 5 Conclusion

In summary, we explored and verified the possibility of using MDSCs-related lncRNA as markers for prognostic analysis of BRCA patients. We successfully validated our model, performing survival analysis, enrichment analysis, TME analysis, TME analysis, TIDE analysis, and drug sensitivity prediction. The results of these studies are conducive to more accurate clinical evaluation and targeted treatment of BRCA patients, and provide new horizons for future research.

## CRediT authorship contribution statement

**Yun Zhong:** Writing – review & editing, Writing – original draft, Visualization, Validation, Supervision, Software, Resources, Project administration, Methodology, Investigation, Formal analysis, Data curation, Conceptualization. **Tong Mu:** Writing – original draft, Formal analysis, Data curation, Conceptualization. **Shunyao Zhang:** Writing – original draft, Formal analysis, Data curation, Conceptualization. **Yuan Xiang:** Writing – original draft, Formal analysis, Data curation, Conceptualization. **Lei Xie:** Writing – original draft, Formal analysis, Data curation, Conceptualization. **Wenxiong Zhang:** Writing – review & editing, Writing – original draft, Visualization, Validation, Supervision, Software, Resources, Project administration, Methodology, Investigation, Formal analysis, Data curation, Conceptualization. **Kang Wang:** Writing – review & editing, Writing – original draft, Visualization, Validation, Supervision, Software, Resources, Project administration, Methodology, Investigation, Funding acquisition, Formal analysis, Data curation, Conceptualization.

## Informed consent

For this type of study formal consent is not required.

## Ethics approval and consent

This article does not contain any studies with human participants or animals performed by any of the authors.

## Availability of data and material

Data is provided within the manuscript or supplementary information files.

## Funding

This study was Project supported by 10.13039/501100004479Jiangxi Provincial Natural Science Foundation (Grant number: 20212BAB206050). Role of the Funding: The funding had no role in the design and conduct of the study; collection, management, analysis, and interpretation of the data; preparation, review, or approval of the manuscript; and decision to submit the manuscript for publication.

## Declaration of competing interest

The authors declare that they have no known competing financial interests or personal relationships that could have appeared to influence the work reported in this paper.

## Figures and Tables

**Figure:**
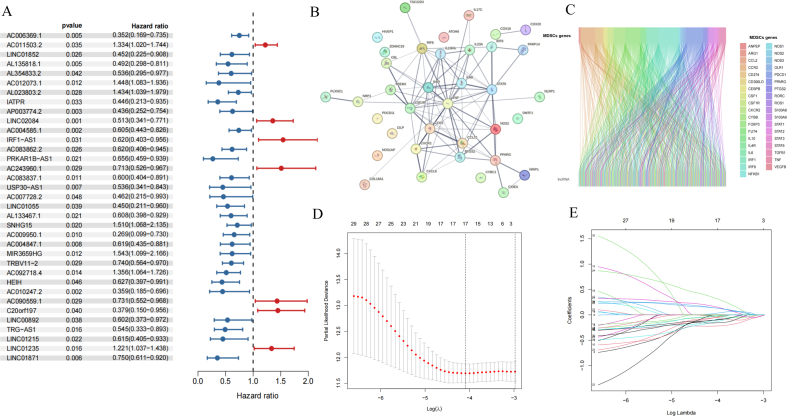
Filter for MDSCs-related lncRNAs. (A) the Forest plot of 35 lncRNAs via univariate COX regression analysis; (B) PPI network among 36 MDSCs-related genes; (C) the Sankey diagram of Coexpression analysis of related lncRNAs and MDSCs genes; (D, E) MDSCs-related prognostic signature was identified via the LASSO COX regression analysis.

**Figure:**
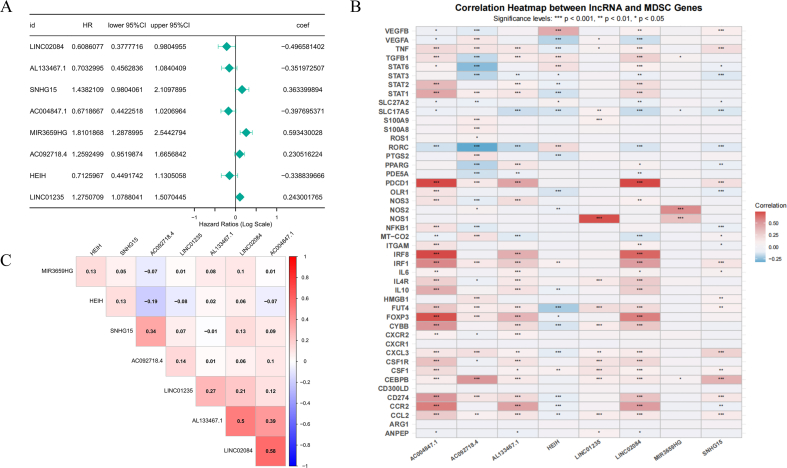
Supplementary figures for analysis of lncRNAs. (A) A forest chart of the 8 lncRNAs we finally filtered out. (B) The correlation heatmap between lncRNAs and MDSCs genes. (C) A heatmap for the relationship among 8 lncRNAs in our model.

**Figure:**
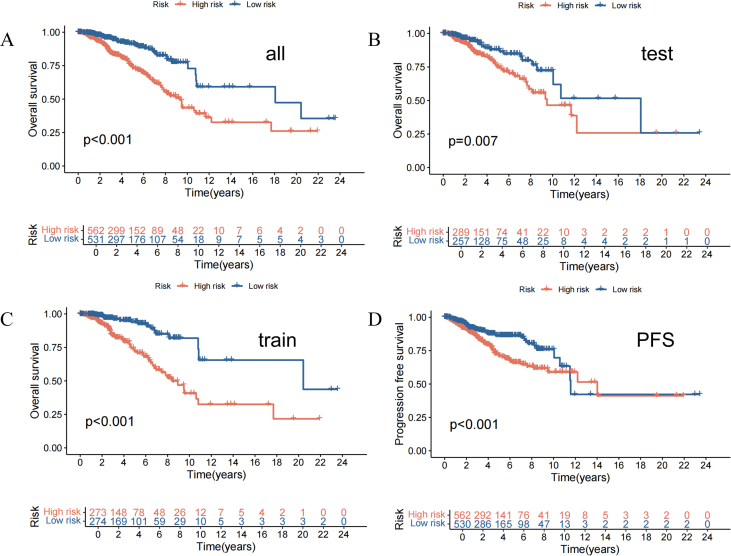
The Kaplan-Meier (K-M) survival curve and of BRCA patients. (A-C) The K-M survival curve for entire group, test group, train group. (D) K-M survival curve of PFS.

**Figure:**
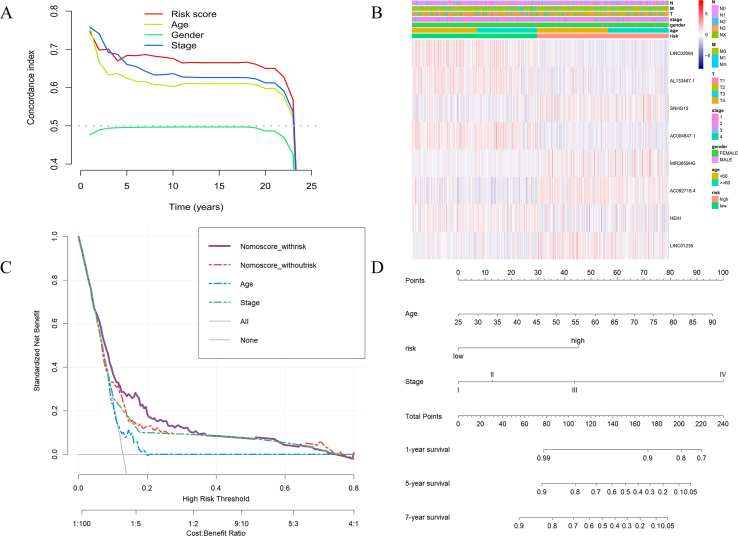
Supplementary figures for clinical heatmap, DCA and nomogram. (A) The concordance index curve of different clinical data and risk score. (B) The clinical heatmap for different clinical characters and 8 lncRNAs in our model. (C) DCA curve for nomoscore (with or without our risk model), age and stage. (D) The empty nomogram for estimating patients' survival probability in 1, 5, 7 years.

**Figure:**
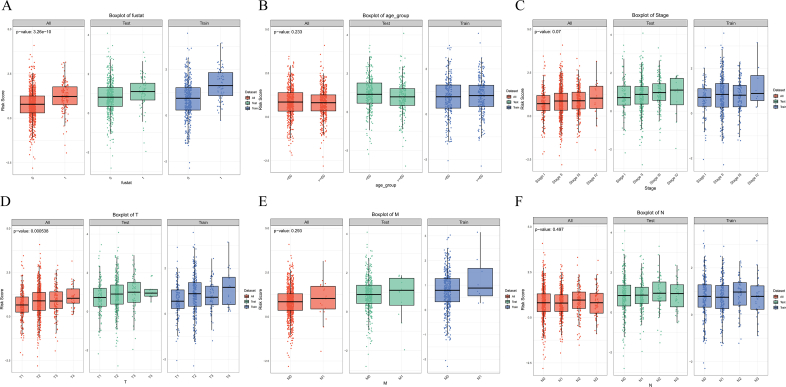
The boxplots for different clinical data and risk score among entire group, test group and train group. (Fustat: 1 = death, 0 = survival).

**Figure:**
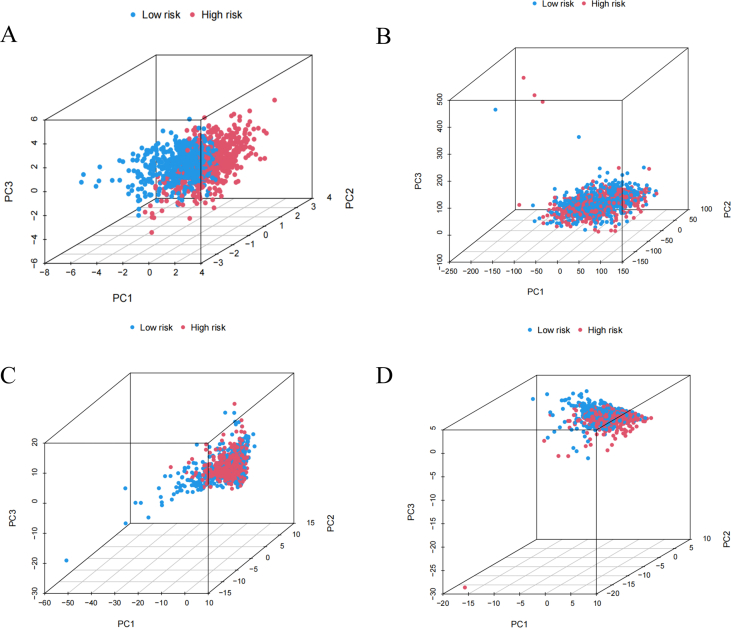
PCA analysis of different biomarkers groups. (A) PCA based on risk signature in our constructed model. (B) PCA based on all gene we selected. (C) PCA based on MDSCs-related lncRNAs. (D) PCA based on MDSCs genes.

**Figure:**
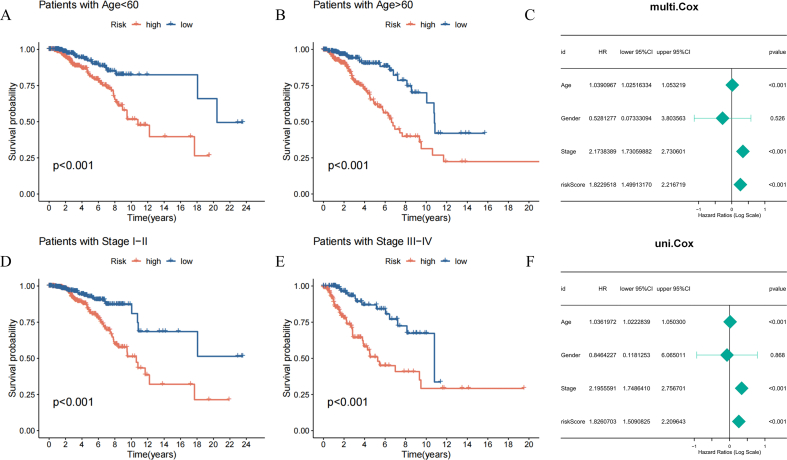
Correlation analysis for different clinicopathological characteristics and independent prognostic analysis. (A, B) Kaplan-Meier survival curves for high-low risk group in age. (D, E) Kaplan–Meier survival curves for high- and low-risk group in stage. The forest plots for multivariate COX (C) and univariate COX (F) regression analysis of age, gender stage and risk score.

**Figure:**
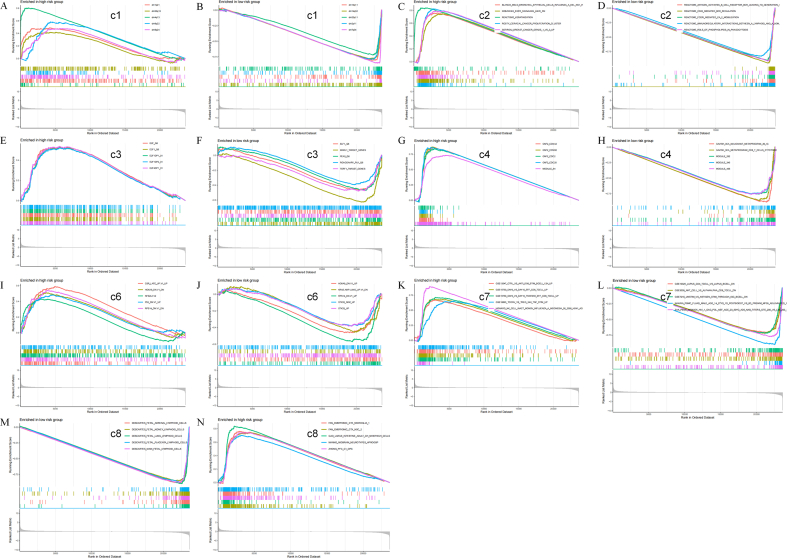
Charts of other gene sets (c1-c4, c6-c8) of GSEA enrichment analysis.

**Figure:**
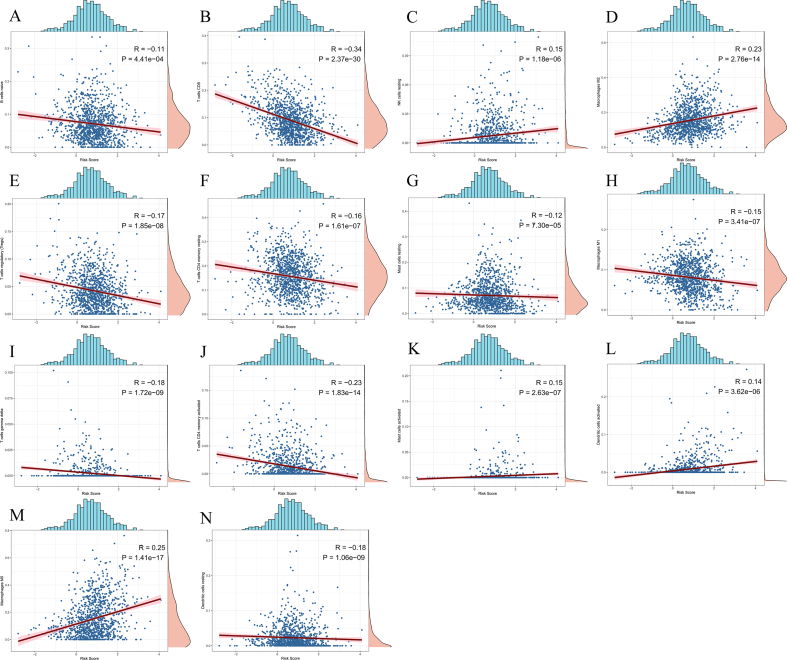
Correlation plot of risk scores with immune cells. (A) B cells naive. (B) T cells CD8. (C) NK cells resting. (D) Macrophages M2. (E) T cells regulatory (Tregs). (F) T cells CD4 memory resting. (G) Mast cells resting. (H) Macrophages M1. (I) T cells gamma delta. (J) T cells CD4 memory activated. (K) Mast cells activated. (L) Dendritic cells activated. (M) Macrophages M0. (N) Dendritic cells resting.

**Figure:**
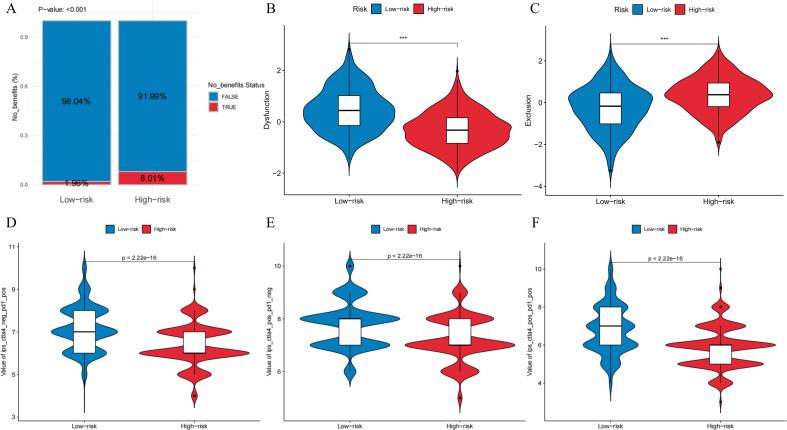
TIDE and IPS. (A) The difference of immunotherapy benefits between two risk groups. (B) Dysfunction score. (C) Exclusion score. (D) ips ctla4 neg pd1 pos score. (E) ips ctla4 pos pd1 neg score. (F) ips ctla4 pos pd1 pos score.

**Figure:**
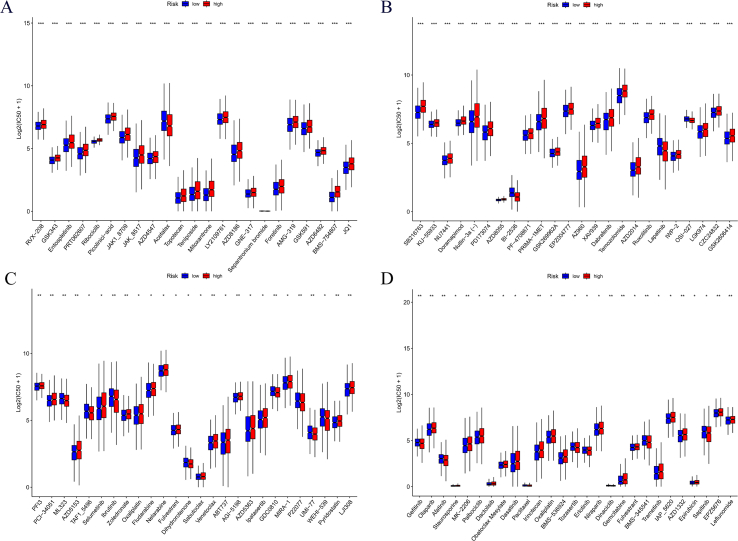
The 94 IC50 significantly different drugs (p < 0.05). (A, B) The 47 IC50-differential drugs with p-value less than 0.001. (C, D) The 47 IC50-differential drugs with p-value between 0.001 and 0,05.

## Data Availability

Data is provided within the manuscript or supplementary information files.

## Abbreviations

AUC: Area Under the Curve; BLCA: Bladder Urothelial Carcinoma; BRCA: Breast Invasive Carcinoma; CC: Cellular Components; CI: Confidence Interval; CMap: Connectivity Map; DCA: Decision Curve Analysis; DESeq2: Differential Expression Analysis for Sequence Count Data; ESTIMATE: Estimation of Stromal and Immune Cells in Malignant Tumor Tissues using Expression Data; FPKM: Fragments Per Kilobase of Transcript per Million Mapped Reads; GDSC: Genomics of Drug Sensitivity in Cancer; GO: Gene Ontology; GSEA: Gene Set Enrichment Analysis; HR: Hazard Ratio; HSP: Heat Shock Protein; ICI: Immune Checkpoint Inhibitor; IC50: Half Maximal Inhibitory Concentration; IPS: Immunophenoscore; KEGG: Kyoto Encyclopedia of Genes and Genomes; K-M: Kaplan-Meier; lncRNAs: Long noncoding RNAs; LASSO: Least Absolute Shrinkage and Selection Operator; MAFTools: Mutation Annotation Format Tools; MF: Molecular Functions; MDSCs: Myeloid-Derived Suppressor Cells; MOA: Mechanism of Action; OS: Overall Survival; PCA: Principal Component Analysis; PFS: Progression-Free Survival; PPI: Protein-Protein Interaction; qPCR: Quantitative Polymerase Chain Reaction; ROC: Receiver Operating Characteristic; RT-qPCR: Reverse Transcription Quantitative Polymerase Chain Reaction; ssGSEA: Single-Sample Gene Set Enrichment Analysis; TCGA: The Cancer Genome Atlas; TIDE: Tumor Immune Dysfunction and Exclusion; TMB: Tumor Mutational Burden; TME: Tumor Microenvironment; TPM: Transcripts Per Million.
