# Supplementary material for: Identification and external validation of a prognostic signature based on myeloid-derived suppressor cells-related LncRNAs to evaluate survival prognosis and treatment efficacy in invasive breast carcinoma
Source: Biochem Biophys Rep. 2025 Sep 16;44:102261. doi: 10.1016/j.bbrep.2025.102261 (PMC12476114; doi:10.1016/j.bbrep.2025.102261)
Supplement: Multimedia component 1 [file mmc1.docx]

**Table S1** Primer sequences for 8 MDSCs-related lncRNAs.

| **Gene id** | **Primer F** | **Primer R** |
| --- | --- | --- |
| AL133467.1 | CCCATCTCCTACAGGTCCCA | AAACTGGGGTTTGGGAGCAA |
| SNHG15 | GCTGAGGTGACGGTCTCAAA | AGCATCTTGGGATTGCTGCT |
| AC004847.1 | CATCTCACCCTCAGCGGTTT | ATGGGGGCCATGGTAGTAGT |
| MIR3659HG | TAGGGGAGGCAGAGTCCTTC | CATCCCCCAGCTCGATACAC |
| AC092718.4 | GGAGGACTCTCCAGTGGCTA | GAGTTAGGATCGTCCGCCTG |
| HEIH | GTGATGCAGCCCCAGTAAGT | GAGCTTCCCTGAAGCCAACT |
| LINC01235 | CAGTGGTCTCAGCTGTGGAG | TGCGTTGACCTGTGAGAGAC |
| LINC02084 | GAACACCAGTGGAAAACGGC | GCGCCCGGTTTGTTTACATT |

**Abbreviation:** MDSCs: Myeloid-derived suppressor cells.
