# Supplementary material for: Identification and external validation of a prognostic signature based on myeloid-derived suppressor cells-related LncRNAs to evaluate survival prognosis and treatment efficacy in invasive breast carcinoma
Source: Biochem Biophys Rep. 2025 Sep 16;44:102261. doi: 10.1016/j.bbrep.2025.102261 (PMC12476114; doi:10.1016/j.bbrep.2025.102261)
Supplement: Multimedia component 2 [file mmc2.docx]

**Table S2** Coef of lncRNAs in LASSO results.

| **id** | **Coef** |
| --- | --- |
| LINC01852 | -0.122090131 |
| AL135818.1 | -0.061535805 |
| AP003774.2 | -0.135231177 |
| LINC02084 | -0.234337035 |
| AC083837.1 | -0.135662657 |
| AL133467.1 | -0.203323623 |
| SNHG15 | 0.223555105 |
| AC009950.1 | -0.169154241 |
| AC004847.1 | -0.105990536 |
| MIR3659HG | 0.431552276 |
| AC092718.4 | 0.1649747 |
| HEIH | -0.182063755 |
| LINC01235 | 0.175800296 |
| LINC01871 | -0.050690142 |
