# Supplementary material for: Identification and external validation of a prognostic signature based on myeloid-derived suppressor cells-related LncRNAs to evaluate survival prognosis and treatment efficacy in invasive breast carcinoma
Source: Biochem Biophys Rep. 2025 Sep 16;44:102261. doi: 10.1016/j.bbrep.2025.102261 (PMC12476114; doi:10.1016/j.bbrep.2025.102261)
Supplement: Multimedia component 3 [file mmc3.docx]

**Table S3** The detailed functions and pathways of 8 gene sets(c1-c8) of GSEA enrichment analysis.

| **Enriched Pathways** |  | **Risk Group** |
| --- | --- | --- |
| **c1.all.v2024.1.Hs.symbols** |  |  |
| chr1q21 | | high |
| chr20q13 | | high |
| chr4q13 | | high |
| chr8q22 | | high |
| chr8q24 | | high |
| chr14q11 | | low |
| chr14q32 | | low |
| chr22q11 | | low |
| chr2p11 | | low |
| chr7q34 | | low |
| **c2.all.v2024.1.Hs.symbols** |  |  |
| BLANCO_MELO_BRONCHIAL_EPITHELIAL_CELLS_INFLUENZA_A_DEL_NS1_INFECTION_DN | | high |
| KOBAYASHI_EGFR_SIGNALING_24HR_DN | | high |
| REACTOME_KERATINIZATION | | high |
| ROSTY_CERVICAL_CANCER_PROLIFERATION_CLUSTER | | high |
| SOTIRIOU_BREAST_CANCER_GRADE_1_VS_3_UP | | high |
| REACTOME_ANTIGEN_ACTIVATES_B_CELL_RECEPTOR_BCR_LEADING_TO_GENERATION_OF_SECOND_MESSENGERS | | low |
| REACTOME_FCERI_MEDIATED_CA_2_MOBILIZATION | | low |
| REACTOME_FCERI_MEDIATED_MAPK_ACTIVATION | | low |
| REACTOME_IMMUNOREGULATORY_INTERACTIONS_BETWEEN_A_LYMPHOID_AND_A_NON_LYMPHOID_CELL | | low |
| REACTOME_ROLE_OF_PHOSPHOLIPIDS_IN_PHAGOCYTOSIS | | low |
| **c3.all.v2024.1.Hs.symbols** |  |  |
| E2F_Q6 | | high |
| E2F1_Q6 | | high |
| E2F1DP1_01 | | high |
| E2F1DP2_01 | | high |
| E2F4DP1_01 | | high |
| ELF1_Q6 | | low |
| MAML1_TARGET_GENES | | low |
| PEA3_Q6 | | low |
| RGAGGAARY_PU1_Q6 | | low |
| TERF1_TARGET_GENES | | low |
| **c4.all.v2024.1.Hs.symbols** |  |  |
| GNF2_CCNA2 | | high |
| GNF2_CCNB2 | | high |
| GNF2_CDC2 | | high |
| GNF2_CDC20 | | high |
| MODULE_54 | | high |
| GAVISH_3CA_MALIGNANT_METAPROGRAM_36_IG | | low |
| GAVISH_3CA_METAPROGRAM_CD8_T_CELLS_CYTOTOXIC | | low |
| MODULE_292 | | low |
| MODULE_345 | | low |
| MODULE_436 | | low |
| **c5.all.v2024.1.Hs.symbols** |  |  |
| GOBP_EPIDERMAL_CELL_DIFFERENTIATION | | high |
| GOBP_EPIDERMIS_DEVELOPMENT | | high |
| GOBP_KERATINIZATION | | high |
| GOBP_KERATINOCYTE_DIFFERENTIATION | | high |
| GOBP_SKIN_DEVELOPMENT | | high |
| GOBP_IMMUNOGLOBULIN_PRODUCTION | | low |
| GOCC_IMMUNOGLOBULIN_COMPLEX | | low |
| GOCC_PLASMA_MEMBRANE_SIGNALING_RECEPTOR_COMPLEX | | low |
| GOCC_T_CELL_RECEPTOR_COMPLEX | | low |
| GOMF_ANTIGEN_BINDING | | low |
| **c6.all.v2024.1.Hs.symbols** |  |  |
| CSR_LATE_UP.V1_UP | | high |
| HOXA9_DN.V1_DN | | high |
| NFE2L2.V2 | | high |
| P53_DN.V1_UP | | high |
| RPS14_DN.V1_DN | | high |
| HOXA9_DN.V1_UP | | low |
| KRAS.AMP.LUNG_UP.V1_DN | | low |
| RPS14_DN.V1_UP | | low |
| STK33_SKM_UP | | low |
| STK33_SKM_UP | | low |
| **c7.all.v2024.1.Hs.symbols** |  |  |
| GSE13547_CTRL_VS_ANTI_IGM_STIM_BCELL_12H_UP | | high |
| GSE15750_DAY6_VS_DAY10_EFF_CD8_TCELL_UP | | high |
| GSE15750_DAY6_VS_DAY10_TRAF6KO_EFF_CD8_TCELL_UP | | high |
| GSE18893_TCONV_VS_TREG_24H_TNF_STIM_UP | | high |
| HOWARD_NK_CELL_INACT_MONOV_INFLUENZA_A_INDONESIA_05_2005_H5N1_AGE_18_49YO_3DY_UP | | high |
| GSE10325_LUPUS_CD4_TCELL_VS_LUPUS_BCELL_DN | | low |
| GSE3039_NKT_CELL_VS_ALPHAALPHA_CD8_TCELL_DN | | low |
| HOEK_PBMC_INACTIVATED_INFLUENZA_ADULT_7DY_UP | | low |
| NAKAYA_PBMC_FLUAD_MALE_AGE_14_27YO_1D_POSTBOOST_VS_0D_PREIMM_MF59_ADJUVANTED_1DY_GENES_IN_BTM_M40_AND_M53_DN | | low |
| ZAK_PBMC_MRKAD5_HIV_1_GAG_POL_NEF_AGE_20_50YO_AD5_NAB_TITERS_GTE_200_VS_LTE_200_1DY_UP | | low |
| **c8.all.v2024.1.Hs.symbols** |  |  |
| FAN_EMBRYONIC_CTX_MICROGLIA_1 | | high |
| FAN_EMBRYONIC_CTX_NSC_2 | | high |
| GAO_LARGE_INTESTINE_ADULT_CH_MKI67HIGH_CELLS | | high |
| MANNO_MIDBRAIN_NEUROTYPES_HPROGBP | | high |
| ZHONG_PFC_C1_OPC | | high |
| DESCARTES_FETAL_ADRENAL_LYMPHOID_CELLS | | low |
| DESCARTES_FETAL_KIDNEY_LYMPHOID_CELLS | | low |
| DESCARTES_FETAL_LUNG_LYMPHOID_CELLS | | low |
| DESCARTES_FETAL_PLACENTA_LYMPHOID_CELLS | | low |
| DESCARTES_MAIN_FETAL_LYMPHOID_CELLS | | low |

**Abbreviation:** GSEA: Gene Set Enrichment Analysis.
