# Supplementary material for: Identification and external validation of a prognostic signature based on myeloid-derived suppressor cells-related LncRNAs to evaluate survival prognosis and treatment efficacy in invasive breast carcinoma
Source: Biochem Biophys Rep. 2025 Sep 16;44:102261. doi: 10.1016/j.bbrep.2025.102261 (PMC12476114; doi:10.1016/j.bbrep.2025.102261)
Supplement: Multimedia component 4 [file mmc4.docx]

**Table S4** The 47 IC50-differential drugs with p-value less than 0.001 and their IC50 (25%-75%).

| **Target Pathways & Drugs** | **High-Risk** |  | **Low-Risk** | **lower-IC50 Risk group** | **P-value** |
| --- | --- | --- | --- | --- | --- |
|  | **IC50 (25%-75%)** |  | **IC50 (25%-75%)** |  |  |
| **Apoptosis regulation** |  |  |  |  |  |
| Sepantronium bromide | 0.01 (0.01-0.02) |  | 0.01 (0.01-0.02) | high | 2.70E-05 |
| **Cell cycle** |  |  |  |  |  |
| BI-2536 | 1.11 (0.7-1.62) |  | 1.59 (1.14-2.33) | high | 1.80E-26 |
| Ribociclib | 50.34 (46.69-54.42) |  | 46.26 (41.82-49.39) | low | 2.20E-35 |
| **Chromatin histone methylation** |  |  |  |  |  |
| EPZ004777 | 180.83 (138.48-244.21) |  | 163.06 (121.83-220.67) | low | 1.00E-04 |
| GSK343 | 18.08 (14.98-21.39) |  | 15.93 (12.87-19.03) | low | 5.40E-12 |
| GSK591 | 104.94 (76.53-152.27) |  | 95.78 (67.45-133.34) | low | 2.50E-04 |
| **Chromatin other** |  |  |  |  |  |
| RVX-208 | 119.15 (96.6-151.7) |  | 110.11 (90.52-136.16) | low | 2.20E-04 |
| JQ1 | 12.76 (8.86-18.17) |  | 10.33 (7.04-14.24) | low | 1.40E-10 |
| **Cytoskeleton** |  |  |  |  |  |
| GSK269962A | 19.7 (16.32-24.33) |  | 18.08 (15.14-22.9) | low | 1.00E-04 |
| **DNA replication** |  |  |  |  |  |
| Temozolomide | 455.27 (333.38-614.17) |  | 359.9 (247.61-516.15) | low | 6.60E-12 |
| Topotecan | 1.33 (0.71-2.43) |  | 1.09 (0.56-1.81) | low | 3.30E-05 |
| Teniposide | 2.02 (0.99-4.14) |  | 1.52 (0.82-2.79) | low | 1.50E-05 |
| Mitoxantrone | 2.29 (1.26-4.29) |  | 1.45 (0.8-2.52) | low | 1.80E-18 |
| **EGFR signaling** |  |  |  |  |  |
| Lapatinib | 20.93 (12.03-33.61) |  | 26.95 (16.66-40.27) | high | 2.80E-07 |
| **ERK MAPK signaling** |  |  |  |  |  |
| Dabrafenib | 115.88 (75.21-177.92) |  | 106.04 (62.37-146.94) | low | 6.40E-05 |
| **Genome integrity** |  |  |  |  |  |
| KU-55933 | 89.25 (73.82-105.86) |  | 82.77 (70.14-98.26) | low | 4.00E-05 |
| NU7441 | 13.96 (10.63-17.86) |  | 12.49 (9.75-16.02) | low | 8.50E-06 |
| **IGF1R signaling** |  |  |  |  |  |
| BMS-754807 | 1.92 (1.19-2.99) |  | 1.15 (0.69-1.88) | low | 6.00E-26 |
| **JNK and p38 signaling** |  |  |  |  |  |
| Doramapimod | 97.97 (83.26-121.99) |  | 88 (76.06-105.81) | low | 4.90E-11 |
| **Metabolism** |  |  |  |  |  |
| GSK2606414 | 45.73 (33.1-64.89) |  | 40.03 (29.82-56.23) | low | 1.00E-05 |
| **Other** |  |  |  |  |  |
| Picolinici-acid | 187.76 (151.75-227.07) |  | 163.82 (129.26-205.47) | low | 8.00E-10 |
| LY2109761 | 178.41 (132.31-243.5) |  | 157.3 (121.06-217.68) | low | 3.60E-04 |
| **Other, kinases** |  |  |  |  |  |
| AZ960 | 8.7 (4.72-15.8) |  | 6.68 (4.12-12.49) | low | 3.40E-04 |
| Ruxolitinib | 139.67 (105.69-175.02) |  | 116.7 (89.57-151.37) | low | 4.80E-10 |
| Entospletinib | 43.18 (31.49-66.83) |  | 37.56 (25.89-54.22) | low | 3.60E-07 |
| PRT062607 | 28.04 (20.44-39.79) |  | 22.82 (17-32.85) | low | 9.10E-11 |
| JAK1_8709 | 68.58 (49.03-98.95) |  | 57.68 (42.31-80.93) | low | 2.80E-08 |
| JAK_8517 | 21.47 (13.21-36.54) |  | 18.06 (10.87-29.72) | low | 6.20E-05 |
| **PI3K/MTOR signaling** |  |  |  |  |  |
| AZD8055 | 0.86 (0.79-0.95) |  | 0.81 (0.73-0.89) | low | 7.40E-14 |
| PF-4708671 | 52.58 (39.2-66.41) |  | 47.53 (36.23-61.28) | low | 1.20E-04 |
| AZD2014 | 8.66 (5.69-14.57) |  | 7.19 (5.02-11.08) | low | 1.10E-05 |
| OSI-027 | 100.67 (90.18-114.05) |  | 109.25 (96.83-122.35) | high | 8.60E-10 |
| CZC24832 | 164 (132.07-206.16) |  | 151.53 (117.76-191.69) | low | 2.80E-05 |
| AZD8186 | 27.36 (17.71-44.04) |  | 22.14 (14.61-37.97) | low | 2.60E-05 |
| GNE-317 | 1.82 (1.28-2.59) |  | 1.6 (1.18-2.29) | low | 9.00E-04 |
| AMG-319 | 137.2 (100.18-188.85) |  | 118.27 (81.15-169.52) | low | 2.30E-06 |
| AZD6482 | 27.47 (22.23-32.95) |  | 24.87 (19.93-29.25) | low | 1.70E-10 |
| **RTK signaling** |  |  |  |  |  |
| PD173074 | 66.35 (45.85-94.58) |  | 55.52 (36.97-77.21) | low | 9.80E-08 |
| AZD4547 | 19.51 (13.69-26.17) |  | 17.37 (12.84-23.84) | low | 9.60E-04 |
| Foretinib | 2.96 (1.74-4.78) |  | 2.38 (1.46-3.96) | low | 9.60E-05 |
| **Unclassified** |  |  |  |  |  |
| Acetalax | 110.44 (63.44-207.41) |  | 144.73 (85.29-252.22) | high | 1.70E-06 |
| **WNT signaling** |  |  |  |  |  |
| SB216763 | 208.12 (152.96-283.73) |  | 152.33 (112.48-212.47) | low | 1.30E-22 |
| XAV939 | 87.08 (67.98-113.49) |  | 78.29 (62.93-97.88) | low | 3.30E-06 |
| IWP-2 | 17.31 (13.81-21.34) |  | 15.88 (12.33-19.46) | low | 2.90E-07 |
| LGK974 | 64.5 (43.85-87.25) |  | 56.61 (40.39-77.82) | low | 3.70E-04 |
| **p53 pathway** |  |  |  |  |  |
| Nutlin-3a (-) | 121.88 (71.3-238.5) |  | 95.07 (53.81-167.15) | low | 8.00E-08 |
| PRIMA-1MET | 111.79 (67.69-189.28) |  | 94.75 (62.06-137.7) | low | 2.10E-05 |

**Abbreviation:** IC50: half maximal inhibitory concentration.
