# Supplementary material for: Identification and external validation of a prognostic signature based on myeloid-derived suppressor cells-related LncRNAs to evaluate survival prognosis and treatment efficacy in invasive breast carcinoma
Source: Biochem Biophys Rep. 2025 Sep 16;44:102261. doi: 10.1016/j.bbrep.2025.102261 (PMC12476114; doi:10.1016/j.bbrep.2025.102261)
Supplement: Multimedia component 5 [file mmc5.docx]

**Table S5** The 46 IC50-differential drugs with p-value between 0.001 and 0.05, and their IC50 (25%-75%).

| **Target Pathways & Drugs** | **High-Risk** |  | **Low-Risk** | **low**er-IC50 Risk group | **P-value** |
| --- | --- | --- | --- | --- | --- |
|  | **IC50 (25%-75%)** |  | **IC50 (25%-75%)** |  |  |
| **Apoptosis regulation** |  |  |  |  |  |
| Obatoclax Mesylate | 4.32 (3.18-5.9) |  | 4.09 (2.94-5.35) | low | 0.013 |
| Sabutoclax | 0.73 (0.5-1.12) |  | 0.7 (0.49-0.99) | low | 0.032 |
| Venetoclax | 9.98 (6.6-14.49) |  | 8.99 (5.93-13.07) | low | 0.005 |
| ABT737 | 10.5 (4.89-19.19) |  | 9.28 (4.53-15.82) | low | 0.014 |
| **Cell cycle** |  |  |  |  |  |
| Palbociclib | 43.72 (25.6-77.88) |  | 40.49 (23.79-64.69) | low | 0.023 |
| Dinaciclib | 0.06 (0.03-0.1) |  | 0.07 (0.04-0.12) | high | 0.001 |
| **Chromatin histone acetylation** |  |  |  |  |  |
| PCI-34051 | 93.59 (69.92-130.03) |  | 88.88 (65.6-115.25) | low | 0.003 |
| **Chromatin histone methylation** |  |  |  |  |  |
| EPZ5676 | 268.87 (201.86-351.33) |  | 252.52 (187.77-328.18) | low | 0.004 |
| **Chromatin other** |  |  |  |  |  |
| PFI3 | 189.65 (163.41-225.65) |  | 187.34 (150.97-218.95) | low | 0.007 |
| AZD5153 | 5.77 (3.33-9.86) |  | 5.27 (3.11-7.71) | low | 0.002 |
| **DNA replication** |  |  |  |  |  |
| Irinotecan | 14.56 (7.77-28.23) |  | 12.68 (6.35-21.97) | low | 0.003 |
| Oxaliplatin | 43.86 (25.99-72) |  | 40.69 (25.14-64.35) | low | 0.036 |
| Gemcitabine | 0.66 (0.23-1.67) |  | 0.47 (0.19-1.22) | low | 0.002 |
| Epirubicin | 0.37 (0.21-0.6) |  | 0.31 (0.2-0.52) | low | 0.01 |
| Leflunomide | 155.59 (116.9-196.11) |  | 149.72 (108.35-188.13) | low | 0.008 |
| Oxaliplatin | 43.86 (25.99-72) |  | 40.69 (25.14-64.35) | low | 0.036 |
| Fludarabine | 164.76 (113.11-227.66) |  | 149.29 (103.75-216.32) | low | 0.022 |
| Nelarabine | 438.5 (323.91-576.07) |  | 413.15 (312.05-548.02) | low | 0.046 |
| **EGFR signaling** |  |  |  |  |  |
| Gefitinib | 24.95 (16.39-35.06) |  | 26.46 (20.1-34.89) | high | 0.003 |
| Afatinib | 6.44 (3.81-9.55) |  | 7.26 (4.8-10.15) | high | 0.002 |
| Erlotinib | 14.37 (9.57-18.84) |  | 14.68 (11.14-18.8) | high | 0.034 |
| Sapitinib | 55.68 (28.27-89.19) |  | 63.04 (37.55-92.89) | high | 0.011 |
| **ERK MAPK signaling** |  |  |  |  |  |
| Trametinib | 2.07 (0.8-4.53) |  | 1.65 (0.73-3.61) | low | 0.012 |
| Selumetinib | 65.39 (35.59-132.12) |  | 58.67 (31.9-106.16) | low | 0.034 |
| **Genome integrity** |  |  |  |  |  |
| Olaparib | 80.13 (55.65-121.88) |  | 73.41 (46.06-111.98) | low | 0.007 |
| Niraparib | 78.39 (50.96-127.02) |  | 71.53 (46.18-112.22) | low | 0.019 |
| **Hormone-related** |  |  |  |  |  |
| Fulvestrant | 18.9 (14.89-24.16) |  | 18.04 (14.06-23.59) | low | 0.038 |
| GDC0810 | 133.3 (105.69-173.78) |  | 143.35 (113.61-182.54) | high | 0.002 |
| **IGF1R signaling** |  |  |  |  |  |
| BMS-536924 | 8.4 (5.07-14.96) |  | 7.58 (4.52-12) | low | 0.007 |
| **Metabolism** |  |  |  |  |  |
| AGI-5198 | 110.56 (90.89-133.58) |  | 104.8 (81.54-129.1) | low | 0.001 |
| **Mitosis** |  |  |  |  |  |
| Paclitaxel | 0.06 (0.02-0.14) |  | 0.06 (0.03-0.15) | high | 0.026 |
| Tozasertib | 17.78 (11.9-26.84) |  | 19.01 (14.17-26.21) | high | 0.041 |
| **Other** |  |  |  |  |  |
| BMS-345541 | 26.84 (17.11-44.84) |  | 30.65 (21.42-45.45) | high | 0.002 |
| IAP_5620 | 182.1 (126.41-267.31) |  | 170.46 (114.44-238.96) | low | 0.023 |
| TAF1_5496 | 46.07 (31.61-64.4) |  | 50.33 (34.68-73.09) | high | 0.02 |
| Zoledronate | 43.46 (33.42-55.09) |  | 40.53 (30.58-53.36) | low | 0.007 |
| **Other, kinases** |  |  |  |  |  |
| Dasatinib | 5.7 (2.58-13.37) |  | 4.58 (2.03-11.13) | low | 0.011 |
| Ibrutinib | 93.63 (47.3-152.8) |  | 110.11 (64.45-163.78) | high | 0.001 |
| AZD5363 | 19.86 (11.25-40.21) |  | 17.58 (9.73-37.75) | low | 0.032 |
| **PI3K/MTOR signaling** |  |  |  |  |  |
| MK-2206 | 23.9 (13.79-41.24) |  | 20.72 (11.82-37.09) | low | 0.004 |
| Dactolisib | 0.23 (0.12-0.43) |  | 0.19 (0.11-0.35) | low | 0.007 |
| Ipatasertib | 35.91 (21.11-57.98) |  | 32.32 (20.37-51.53) | low | 0.042 |
| **Protein stability and degradation** |  |  |  |  |  |
| ML323 | 87.62 (65.19-116.94) |  | 97.93 (73.61-128.6) | high | 0.001 |
| **RTK signaling** |  |  |  |  |  |
| Staurosporine | 0.05 (0.03-0.12) |  | 0.04 (0.02-0.09) | low | 0.039 |
| AZD1332 | 49.58 (32.06-76.58) |  | 45.87 (27.88-67.92) | low | 0.005 |
| **Unclassified** |  |  |  |  |  |
| Dihydrorotenone | 2.34 (1.73-3.18) |  | 2.6 (1.85-3.52) | high | 0.012 |

**Abbreviation:** IC50: half maximal inhibitory concentration.
