# Supplementary material for: Identification and external validation of a prognostic signature based on myeloid-derived suppressor cells-related LncRNAs to evaluate survival prognosis and treatment efficacy in invasive breast carcinoma
Source: Biochem Biophys Rep. 2025 Sep 16;44:102261. doi: 10.1016/j.bbrep.2025.102261 (PMC12476114; doi:10.1016/j.bbrep.2025.102261)
Supplement: Multimedia component 6 [file mmc6.docx]

**Table S6** The 96 no obviously sensitive drugs with p-value above 0.05 and their IC50 (25%-75%).

| **Target Pathways & Drugs** | **High-Risk** |  | **Low-Risk** | **low**er-IC50 Risk group | **P-value** |
| --- | --- | --- | --- | --- | --- |
|  | **IC50 (25%-75%)** |  | **IC50 (25%-75%)** |  |  |
| **ABL signaling** |  |  |  |  |  |
| Nilotinib | 41.45 (27.08-63.22) |  | 39.96 (26.65-55.73) | low | 0.082 |
| **Apoptosis regulation** |  |  |  |  |  |
| Navitoclax | 8.79 (4.09-16.49) |  | 8.14 (3.67-14.83) | low | 0.156 |
| LCL161 | 152.62 (113.06-199.98) |  | 148.86 (113.08-190.66) | low | 0.305 |
| AZD5582 | 10.32 (4.99-18.57) |  | 11.46 (5.88-19.5) | high | 0.203 |
| AZD5991 | 85.23 (40.47-147.7) |  | 88.84 (52.74-146.5) | high | 0.19 |
| MIM1 | 50.74 (33.98-71.2) |  | 50.88 (33.56-70.92) | high | 0.994 |
| **Cell cycle** |  |  |  |  |  |
| AZD7762 | 1.12 (0.53-2.26) |  | 1.09 (0.55-2.13) | low | 0.922 |
| Wee1 Inhibitor | 7.75 (4.84-12.82) |  | 8.43 (5.65-12.85) | high | 0.148 |
| RO-3306 | 21.23 (19.48-23.27) |  | 21.38 (19.61-23.28) | high | 0.748 |
| MK-1775 | 1.81 (1.09-3.21) |  | 2.01 (1.21-3.11) | high | 0.097 |
| AZD5438 | 8.91 (5.21-14.96) |  | 8.98 (5.98-15.71) | high | 0.11 |
| CDK9_5576 | 0.62 (0.4-1.02) |  | 0.68 (0.44-1.06) | high | 0.119 |
| CDK9_5038 | 0.08 (0.05-0.17) |  | 0.1 (0.06-0.18) | high | 0.051 |
| MK-8776 | 30.21 (15.95-48.95) |  | 27.21 (13.85-44.11) | low | 0.076 |
| **Chromatin histone acetylation** |  |  |  |  |  |
| Vorinostat | 4.13 (3.07-5.58) |  | 4.23 (3.29-5.78) | high | 0.066 |
| Entinostat | 10.01 (6.54-15.25) |  | 9.04 (5.73-14.26) | low | 0.053 |
| OF-1 | 61.26 (45-84.31) |  | 60.98 (45.34-85.11) | low | 0.891 |
| **Chromatin other** |  |  |  |  |  |
| I-BET-762 | 28.28 (18.12-44.95) |  | 26.76 (18.3-41.75) | low | 0.347 |
| OTX015 | 12.11 (6.19-25.3) |  | 12.51 (6.99-23.27) | high | 0.483 |
| I-BRD9 | 80.95 (55.77-118.73) |  | 87.77 (59.66-118.68) | high | 0.166 |
| **Cytoskeleton** |  |  |  |  |  |
| PAK_5339 | 10.99 (9.02-13.18) |  | 11.14 (9.32-13.4) | high | 0.302 |
| BDP-00009066 | 10.41 (7.46-15.37) |  | 10.14 (7.83-14.37) | low | 0.8 |
| **DNA replication** |  |  |  |  |  |
| Camptothecin | 0.1 (0.05-0.19) |  | 0.09 (0.05-0.15) | low | 0.061 |
| Cisplatin | 25.79 (13.14-54.95) |  | 25.72 (12.86-52.71) | low | 0.531 |
| Cyclophosphamide | 179.7 (139.09-224.46) |  | 177.55 (137.69-226.09) | low | 0.729 |
| **EGFR signaling** |  |  |  |  |  |
| AZD3759 | 14.25 (10.65-19.65) |  | 15.08 (11.32-19.46) | high | 0.198 |
| Osimertinib | 5.96 (3.43-9.24) |  | 6.41 (4.18-9.19) | high | 0.116 |
| **ERK MAPK signaling** |  |  |  |  |  |
| PLX-4720 | 90.37 (55.28-158.37) |  | 83.27 (55.38-133.53) | low | 0.079 |
| PD0325901 | 1.73 (0.86-3.23) |  | 1.58 (0.88-2.86) | low | 0.168 |
| SCH772984 | 13.52 (7.14-25.47) |  | 13.73 (8.14-23.93) | high | 0.444 |
| ERK_2440 | 13.27 (7.41-25.22) |  | 13.82 (7.93-24.96) | high | 0.564 |
| ERK_6604 | 28.06 (16.46-50.66) |  | 27.88 (17.89-49.44) | low | 0.462 |
| KRAS (G12C) Inhibitor-12 | 85.26 (54.52-125.83) |  | 86.33 (56.92-122.07) | high | 0.654 |
| Ulixertinib | 15.79 (10.75-23.22) |  | 16.07 (12.09-24.36) | high | 0.159 |
| VX-11e | 17.8 (10.21-31.83) |  | 17.57 (10.87-30.69) | low | 0.699 |
| **Genome integrity** |  |  |  |  |  |
| Mirin | 117.62 (75.68-190.67) |  | 117.75 (81.91-178.58) | high | 0.964 |
| Talazoparib | 26.41 (11.19-55.9) |  | 24.43 (10.95-52.41) | low | 0.306 |
| VE-822 | 29.63 (17.68-49.4) |  | 32.38 (17.98-47.93) | high | 0.745 |
| AZD6738 | 7.53 (4.33-13.01) |  | 8.09 (4.88-13.07) | high | 0.19 |
| Telomerase Inhibitor IX | 1.67 (1.05-2.69) |  | 1.71 (1.19-2.77) | high | 0.162 |
| BIBR-1532 | 142.44 (108.55-185.28) |  | 140.4 (111.51-180.07) | low | 0.873 |
| VE821 | 67.62 (40.61-101.44) |  | 64.34 (42.02-102.94) | low | 0.726 |
| **Hormone-related** |  |  |  |  |  |
| Tamoxifen | 38.65 (29.18-49.35) |  | 37.26 (28.6-46.68) | low | 0.066 |
| **IGF1R signaling** |  |  |  |  |  |
| GSK1904529A | 76.39 (54.33-108.21) |  | 78.48 (57.54-105.59) | high | 0.533 |
| Linsitinib | 44.01 (28.66-64.57) |  | 43.15 (31.33-62.51) | low | 0.375 |
| IGF1R_3801 | 5.04 (2.15-12.75) |  | 4.84 (2.17-11) | low | 0.809 |
| NVP-ADW742 | 15.53 (9.93-25.28) |  | 14.73 (10.35-23.03) | low | 0.475 |
| **Metabolism** |  |  |  |  |  |
| Daporinad | 0.01 (0.01-0.03) |  | 0.01 (0.01-0.03) | low | 0.728 |
| AGI-6780 | 61.46 (48.03-80.54) |  | 64.62 (51.48-82.37) | high | 0.093 |
| **Mitosis** |  |  |  |  |  |
| Vinblastine | 0.02 (0.01-0.05) |  | 0.02 (0.01-0.05) | low | 0.641 |
| Docetaxel | 0.01 (0.01-0.02) |  | 0.01 (0.01-0.02) | high | 0.488 |
| ZM447439 | 19.83 (14.04-27.96) |  | 20.1 (14.85-26.83) | high | 0.645 |
| Alisertib | 7.01 (3.14-17.29) |  | 5.96 (2.88-13.61) | low | 0.106 |
| Docetaxel | 0.01 (0.01-0.02) |  | 0.01 (0.01-0.02) | high | 0.488 |
| Vinorelbine | 0.05 (0.02-0.14) |  | 0.05 (0.02-0.12) | low | 0.195 |
| **Other** |  |  |  |  |  |
| Cytarabine | 5.61 (2.86-11.76) |  | 5.93 (2.67-10.67) | high | 0.455 |
| 5-Fluorouracil | 111.5 (57.54-241.94) |  | 122.46 (58.78-204.67) | high | 0.815 |
| YK-4-279 | 9.09 (4.44-19.94) |  | 9.11 (4.65-19.24) | high | 0.958 |
| Pevonedistat | 2.3 (1.03-4.75) |  | 2.53 (1.29-4.78) | high | 0.361 |
| Eg5_9814 | 0.05 (0.02-0.08) |  | 0.05 (0.03-0.07) | high | 0.341 |
| VSP34_8731 | 10.73 (7.61-14.91) |  | 11.05 (8.49-14.72) | high | 0.072 |
| Dactinomycin | 0.09 (0.05-0.14) |  | 0.08 (0.05-0.13) | low | 0.462 |
| BPD-00008900 | 96.6 (69.53-136.64) |  | 93.48 (68.58-124.83) | low | 0.17 |
| **Other, kinases** |  |  |  |  |  |
| Sorafenib | 15.66 (10.67-22.55) |  | 14.98 (10.81-20.45) | low | 0.147 |
| AZD1208 | 205.86 (160.88-258.52) |  | 198.44 (156.8-246.15) | low | 0.152 |
| WZ4003 | 43.92 (28.03-68.67) |  | 41.15 (28.39-61.26) | low | 0.071 |
| IRAK4_4710 | 143.38 (116.69-175.3) |  | 143.99 (119.84-171.3) | high | 0.772 |
| ULK1_4989 | 10.04 (6.45-17.01) |  | 10.26 (6.8-15.92) | high | 0.967 |
| GSK2578215A | 139.87 (108.46-173.85) |  | 141.27 (110.88-170.24) | high | 0.887 |
| **PI3K/MTOR signaling** |  |  |  |  |  |
| Pictilisib | 3.94 (2.3-7.22) |  | 3.44 (2.11-6.67) | low | 0.096 |
| Rapamycin | 0.11 (0.07-0.17) |  | 0.11 (0.07-0.17) | low | 0.22 |
| Uprosertib | 21.9 (11.97-37.44) |  | 18.92 (11.35-34.8) | low | 0.174 |
| Alpelisib | 35.77 (21.15-62.41) |  | 31.88 (20.57-56.27) | low | 0.222 |
| Taselisib | 8.44 (3.89-17.36) |  | 6.73 (3.56-15.62) | low | 0.188 |
| Buparlisib | 2.57 (1.85-3.63) |  | 2.47 (1.86-3.65) | low | 0.51 |
| Afuresertib | 13.72 (7.31-24.38) |  | 11.97 (6.95-21.52) | low | 0.102 |
| Uprosertib | 21.9 (11.97-37.44) |  | 18.92 (11.35-34.8) | low | 0.174 |
| AT13148 | 39.62 (21.42-72.69) |  | 35.27 (22.44-67.89) | low | 0.232 |
| **Protein stability and degradation** |  |  |  |  |  |
| Bortezomib | 0.01 (0.01-0.01) |  | 0.01 (0.01-0.01) | high | 0.12 |
| Luminespib | 0.09 (0.05-0.19) |  | 0.09 (0.05-0.18) | high | 0.877 |
| MG-132 | 0.21 (0.16-0.27) |  | 0.21 (0.17-0.27) | high | 0.951 |
| **RTK signaling** |  |  |  |  |  |
| Axitinib | 22.05 (17.25-29.49) |  | 22.37 (18-28.41) | high | 0.508 |
| Crizotinib | 26.08 (16.66-40.22) |  | 24.91 (17.35-35.91) | low | 0.307 |
| SB505124 | 8.26 (5.66-12.65) |  | 7.96 (5.73-11.68) | low | 0.35 |
| Cediranib | 8.36 (6.02-12.53) |  | 8.18 (6.16-11.53) | low | 0.637 |
| Savolitinib | 13.75 (10.23-17.93) |  | 13.6 (10.27-17.72) | low | 0.692 |
| **Unclassified** |  |  |  |  |  |
| Podophyllotoxin bromide | 0.53 (0.35-0.9) |  | 0.54 (0.36-0.9) | high | 0.87 |
| Gallibiscoquinazole | 13.58 (10.7-16.77) |  | 13.66 (11.07-16.82) | high | 0.503 |
| Elephantin | 30.51 (20.11-49.93) |  | 31.09 (20.3-47.03) | high | 0.913 |
| Carmustine | 435.86 (342.36-603.58) |  | 469.97 (349.48-612.08) | high | 0.205 |
| Vincristine | 0.17 (0.07-0.42) |  | 0.17 (0.08-0.42) | high | 0.628 |
| Sinularin | 34.32 (25.42-50.3) |  | 36.43 (26.68-48.52) | high | 0.459 |
| **WNT signaling** |  |  |  |  |  |
| Wnt-C59 | 69.02 (53.84-87.34) |  | 72.31 (54.93-90.36) | high | 0.217 |
| MN-64 | 111.13 (83.92-146.24) |  | 111.39 (87.53-151.73) | high | 0.476 |
| WIKI4 | 39.02 (31.42-47.19) |  | 39.05 (33.07-45.99) | high | 0.269 |
| AZ6102 | 11.85 (8.25-17.85) |  | 11.06 (8.43-15.85) | low | 0.153 |

**Abbreviation:** IC50: half maximal inhibitory concentration.
